# Ultra-long-range spin coupling in graphene revealed by atomically resolved spin excitations

**DOI:** 10.1038/s41467-026-72624-0

**Published:** 2026-05-19

**Authors:** Beatriz Viña-Bausá, António Tavares Costa, Joao Henriques, Eva Cortés-del Río, Roberto Carrasco, Pierre Mallet, Jean-Yves Veuillen, Joaquín Fernández-Rossier, Iván Brihuega

**Affiliations:** 1 Departamento de Física de la Materia Condensada, Universidad Autónoma de Madrid, Madrid, Spain; 2 International Iberian Nanotechnology Laboratory (INL); Avenida Mestre José Veiga, Braga, Portugal; 3 Physics Center of Minho and Porto Universities (CF-UM-UP), Universidade do Minho; Campus de Gualtar, Braga, Portugal; 4 Universidade de Santiago de Compostela, Santiago de Compostela, Spain; 5 Department of Physics, University of Hamburg, Hamburg, Germany; 6 Université Grenoble Alpes, Grenoble, France; 7 CNRS, Institut Néel, Grenoble, France; 8 Condensed Matter Physics Center (IFIMAC) and Instituto Nicolás Cabrera (INC), Universidad Autónoma de Madrid, Madrid, Spain

**Keywords:** Magnetic properties and materials, Electronic properties and devices, Electronic properties and devices, Two-dimensional materials, Electronic properties and materials

## Abstract

Magnetic interactions between localized spins-½ play a central role in quantum magnetism, spin-based quantum computing, and quantum simulation. The range and strength of these interactions are key figures of merit. Here, we probe exchange interactions in pairs of spins-½ introduced by chemisorption of individual hydrogen atoms on graphene. Using scanning tunneling microscopy and inelastic electron tunneling spectroscopy, supported by large-scale mean-field Hubbard calculations, we demonstrate 3 meV exchange couplings at separations beyond 10 nm, surpassing all prior systems. The couplings can be ferro- or antiferromagnetic depending on the relative sublattice arrangement. Real-space mapping of spin excitation amplitudes enables characterization with atomic-resolution. Through atomic manipulation we extend this control to spin trimers, revealing collective spin excitations when pairwise exchange couplings are comparable.

## Abstract (web summary)

Researchers show ultra-long-range interactions between atomic spins on graphene, reporting meV coupling beyond 10 nm. Controlling their arrangement with atomic scale precision they tune interactions and build few-spin systems with collective behavior.

## Introduction

Understanding and controlling magnetic coupling at a fundamental level is crucial for spin-based quantum technologies^1–3^. In this context, research efforts in the last decades have focused on enhancing spin lifetimes, coherence times and, importantly, on pushing the limits of the spatial and energy scales^3–9^. Different platforms have been investigated, including semiconductor quantum dots^10–12^; electronic and nuclear spins of individual dopants in bulk, solid-state systems^13–15^ or electron spins provided by single atoms or molecules on surfaces^16,17^. However, it still remains a challenge to combine in the same system atomic-scale control with sufficiently strong long-range interactions that provide thermal stability. For instance, for artificial structures made with atomic manipulation of *d* and *f* transition metal atoms on surfaces, the range of magnetic coupling is restricted to the atomic scale, and exchange energies are of the order of a few meV^18,19^. More recently, carbon-based molecular spin systems obtained via on-surface synthesis have gained attention because of their chemical versatility, weak spin-orbit coupling and larger exchange energies^20–26^. Still, in these nanoscale systems, exchange with the conducting surface is expected to limit the spin lifetimes^19,27^.

Here, we study a S = 1/2 point-defect, atomic hydrogen chemisorbed in graphene^28^, whose magnetic moment extends, and strongly interacts, significantly beyond the atomic scale. Atomically modified graphene constitutes a sweet spot between solid-state and atom-by-atom approaches, where long-range interactions coexist with large exchange energies. Together with this, our system also combines two exceptional peculiarities: we can have coexisting ferromagnetic and antiferromagnetic interactions, and our platform enables carrying out atomic-scale manipulations. With all this, we demonstrate that graphene provides a unique playground to understand, explore, and control the interactions between S = 1/2 magnetic moments.

By Scanning Tunneling Microscopy/Spectroscopy (STM/STS), we build and characterize our magnetic configurations: we use the STM tip to selectively introduce S = 1/2 magnetic moments by attaching single H atoms to graphene. Our atomically resolved inelastic electron tunneling spectroscopy (IETS) data probes the spin excitations of pairs and trios of H atoms on graphene. Combined with large-scale mean-field Hubbard (MFH) and Random Phase Approximation (RPA) calculations, these results provide a systematic study as well as real-space visualization and quantification of the exchange interactions as a function of distance and sublattice.

## Inducing S = 1/2 magnetic moments in graphene with single H atoms

Our work relies on the fact that an individual H atom covalently attached to graphene leaves an unpaired electron and therefore induces a S = 1/2 magnetic moment^28–30^. When the C-H bond is formed, both the carbon *p*_*z*_ orbital and one electron from the π cloud are taken away from graphene. As a result, a quasilocalized state emerges at the Dirac point, known as zero mode^29^. Because of Coulomb repulsion, double occupation of this state is blocked, resulting in the formation of a local moment. The density of states (DOS) of a single H atom in graphene is characterized by the presence of two narrow peaks at the Fermi level (E_F_), whose energy splitting, in the range of 10 meV, is a metric of the double occupation energy overhead, that slightly depends on the local environment^28^, on account of the quasi-localized nature of the state^29^. These peaks correspond to the addition and removal of an electron from the quasi-localized state, relative to the singly occupied state.

Magnetic moments induced in graphene by H atoms have been observed to extend over several nanometers, exhibiting threefold symmetry dictated by the graphene crystal structure^28^. In this way, on STM images, single H atoms can be identified because they appear as bright protrusions and are surrounded by a threefold, $\sqrt{3}{{{\rm{x}}}}\sqrt{3}$ pattern rotated 30° with respect to the graphene lattice. The corresponding adsorption sublattice can be inferred because this threefold modulation associated to the magnetic state points in opposite (parallel) directions for H atoms positioned on opposite (equivalent) sublattices. The magnetic moment is essentially induced only on the graphene sublattice opposite to the H adsorption site.

The possibility of carrying out atomic-scale manipulations has also been demonstrated^28,31^ and constitutes a fundamental pillar for this work. Using the STM, H atoms can be selectively attached and removed in graphene.

## Results and Discussion

### Two interacting S = 1/2 spins

We start by building the simplest system that hosts inelastic spin excitations at zero magnetic field: two interacting S = 1/2 magnetic moments. We accomplish this by arranging well-isolated pairs of H atoms on graphene, aiming to avoid the presence of other adsorbed H atoms at distances less than 10 nanometers. The spin of the ground state of H dimers is determined by the relative sublattice (A or B) adsorption site of the H atoms^28,30^ in agreement with Lieb’s theorem^32^. When both H atoms are adsorbed on opposite graphene sublattices (AB pair), the two induced S = 1/2 couple antiferromagnetically and have a singlet ground state, with S = 0. At short separations (≤ 1.5 nm), the AB pair adopts a closed-shell singlet (S = 0) state due to strong orbital hybridization^33^. As the AB distance increases, a smooth transition from closed to open occurs. Conversely, for two H atoms adsorbed on the same graphene sublattice (AA pairs), the exchange coupling is ferromagnetic, and the ground state is an open-shell triplet (S = 1), regardless of separation. Given the very small spin-orbit coupling in graphene, magnetic anisotropy is negligible and such triplet state is degenerate at zero magnetic field^34^.

According to the selection rule imposed by the conservation of angular momentum, possible spin excitations should satisfy ΔS = 0 or ΔS = ± 1^18^. For AB pairs, with a singlet ground state (S = 0), spin transitions to the triplet excited state (S = 1) can be induced by inelastic tunneling electrons, while the AA pair will have a triplet ground state (S = 1) with possible transitions to the singlet excited state (S = 0). Thus, pairs of chemisorbed hydrogen atoms in graphene permit us to create exchange-coupled S = 1/2 pairs with both ferro and antiferromagnetic coupling, and, either S = 0 or S = 1 ground states.

**Fig. 1:**
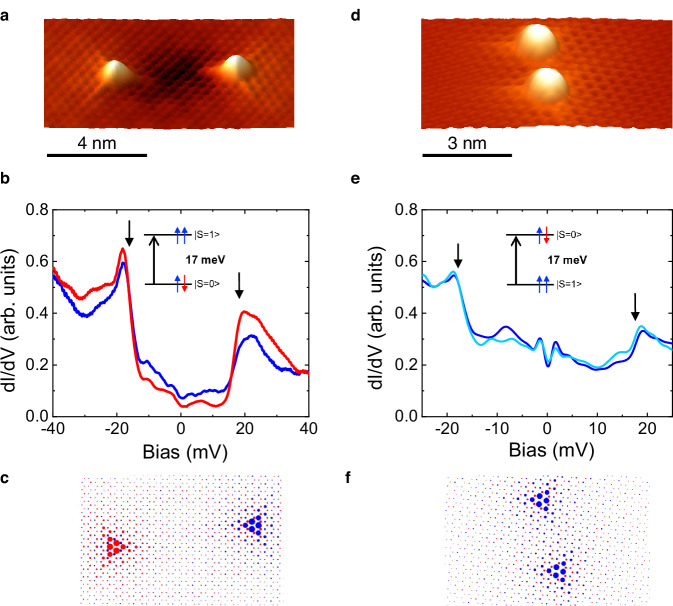
Two interacting S = 1/2 spins. **a** Topography image of 2H atoms adsorbed on opposite graphene sublattices (AB pair) at 4.4 nm (4 mV, 0.05 nA). **b** *dI/dV* spectra measured on each of the H atoms (50 mV, 0.1 nA). The red (blue) curve has been measured on the left (right) atom. Arrows indicate the inelastic steps that correspond to the spin excitations. The singlet-to-triplet transition energy is 17 meV. **c** MFH atomic magnetization of the AB configuration. Red and blue colors indicate opposite spin orientation. **d** Topography image of 2H atoms adsorbed on the same graphene sublattices (AA pair) at 2.4 nm (30 mV, 0.1 nA). **e** *dI/dV* spectra measured on each of the H atoms (30 mV, 0.1 nA). The dark (light) blue curve has been measured on the top (bottom) atom. Arrows indicate the inelastic steps that correspond to the spin excitations. The triplet-to-singlet transition energy is 17 meV**. f** MFH atomic magnetization of the AA configuration. Red and blue colors indicate opposite spin orientation.

Our results for an antiferromagnetically coupled AB pair, separated by 4.4 nm, are shown in Fig. 1a–c. The spectra, measured with a superconducting tip and here deconvoluted (see also Supplementary Tables 1 and 2), present clear energy-symmetric steps at both bias polarities, indicated with black arrows, with a large increase in the differential conductance. This is the fingerprint^18,23^ of spin excitations in inelastic electron tunneling spectroscopy (IETS). The spin excitation takes place at 17 meV and corresponds to the transition from the singlet ground state to the triplet excited state (see methods for details). We have included a scheme of the energy levels to illustrate this. Additionally, Fig. 1c shows a MFH calculation of the atomic magnetization for this precise AB configuration, where red and blue indicate opposite spin directions, confirming the antiferromagnetic ground state of the AB configuration.

The analogous ferromagnetic situation is illustrated in Fig. 1d–f. Here, we arrange an AA pair at 2.4 nm to create a system with a ground state of total spin S = 1 (Fig. 1d). Again, we observe inelastic features at 17 meV, indicated with arrows (Fig. 1e), corresponding to the triplet to singlet excitation of such a configuration and a ferromagnetic ground state in the MFH magnetization calculation (Fig. 1f).

In the AA case, in addition to the symmetric inelastic transitions, we systematically observe spectral features at low energy, similar to those of the monomers. In this regard, AA pairs are a unique system, where the addition energies are smaller than the inelastic spin excitation energies. Beyond this addition-energy voltage, the occupation of the hydrogen-induced zero modes is fluctuating between 1 and either 0 (negative bias) or 2 (positive bias). Therefore, at that bias, there is still a finite probability that the AA pair behaves like a S = 1/2 dimer. Additional data of H pairs and their *dI/dV* spectra can be found on the extensive dataset presented in Supplementary Tables 1 and 2.

#### Control over the magnetic coupling

**Fig. 2:**
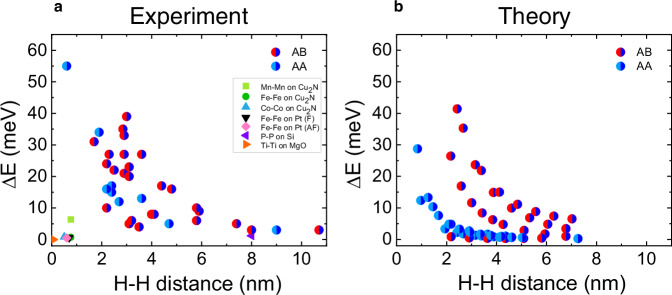
Distance dependence of magnetic coupling. **a** Experimental (IETS) excitation energy for AA and AB pairs as a function of the distance between H atoms. Literature exchange values have been included for comparison from refs. ^18,36–40^. **b** MFH calculation of the excitation energy for AA and AB pairs as a function of the distance between H atoms. Red-blue (blue-blue) dots correspond to AB (AA) pairs.

We have systematically arranged many different pairs of H atoms on graphene, both of AA and AB type, performed IETS measurements and extracted the spin excitation energies (Supplementary Tables 1 and 2 and Supplementary Fig. 1-2). In Fig. 2a we plot the experimental excitation energy as a function of the distance between H atoms for different configurations. The measured values are compared with MFH calculations (see Fig. 2b), which show the energy difference between the excited and ground state for both AA and AB pairs in different relative positions lying on graphene crystallographic directions. The calculations account for the three main experimental findings: first, interactions observable with IETS, i.e., with more than 3 meV, are observed at separations of up to 10 nm. Second, for the same distance, coupling in AA pairs is most often weaker than in AB ones. Third, the strength of the exchange coupling does not only depend on distance, but also on the relative orientation of the anisotropically induced magnetic moments (see SI4). Therefore, the controlled chemisorption of atomic hydrogen on graphene permits the creation of S=1/2 pairs with three different knobs to tune the strength and sign of their exchange interactions: distance, sublattice, and orientation relative to the crystallographic axis.

Several exchange mechanisms are at play. First, direct exchange, relevant for AA pairs, for which the zero-modes overlap in real space, which promotes ferromagnetic interaction. Second, kinetic exchange, relevant for AB pairs, that promotes antiferromagnetic interaction. Third, indirect exchange (RKKY-like) interactions, both for AA and AB cases, mediated by the electrons in the extended states that couple with the local moments induced by the chemisorbed hydrogens. Since local moments are induced in one sublattice only, and the sign of the RKKY interaction in graphene is FM (AF) for AA (AB) pairs, RKKY coupling also contributes to establish the interplay between the sign of the interaction and the sublattice degree of freedom^35^. Although it is difficult to quantitatively disentangle the contributions of the different exchange mechanisms, we find that kinetic and direct exchange dominate in AB and AA pairs, respectively. For AB pairs, the full excitation energy decays as the inverse of the effective on-site interaction, characteristic of kinetic exchange. Moreover, an effective Hubbard dimer model reproduces the same angular dependence as the full calculation. For AA pairs, the spatial decay of the excitation energy is consistent with direct exchange.

The range and strength of spin interactions between H induced moments is dramatically larger than in any other platform, as shown in Fig. 2a. where we have included the literature values for exchange couplings in different paired atomic systems (Supplementary Fig. 3 for more details).

Our length scales are one order of magnitude larger than those required in *d* and *f* shells coupled by direct exchange^18,36–39^ and, in diradical nanographenes, with exchange interactions in the order of 20 meV, the separation of local moments is at least 5 times smaller than in our case^23,24^. Moreover, the only anticipated competing case with exchange interactions between the electronic spins at comparable long distances are two donors in Silicon, relevant for quantum computing, with theoretical predicted values^13,40^ in the range of μeV at a distance of 9 nm, to be compared with the 5 meV in our case. We also note that hypothetical metamaterials built with an artificial lattice of chemisorbed H atoms in graphene^41^ would have exchange energies of the order of 20 meV, so that collective magnetic states would be robust at room temperature.

All these extensive experiments and calculations presented in Fig. 2 provide a complete picture of the energy scale of interactions between two S = 1/2 magnetic moments in graphene.

#### Spatially mapping spin excitations

**Fig. 3:**
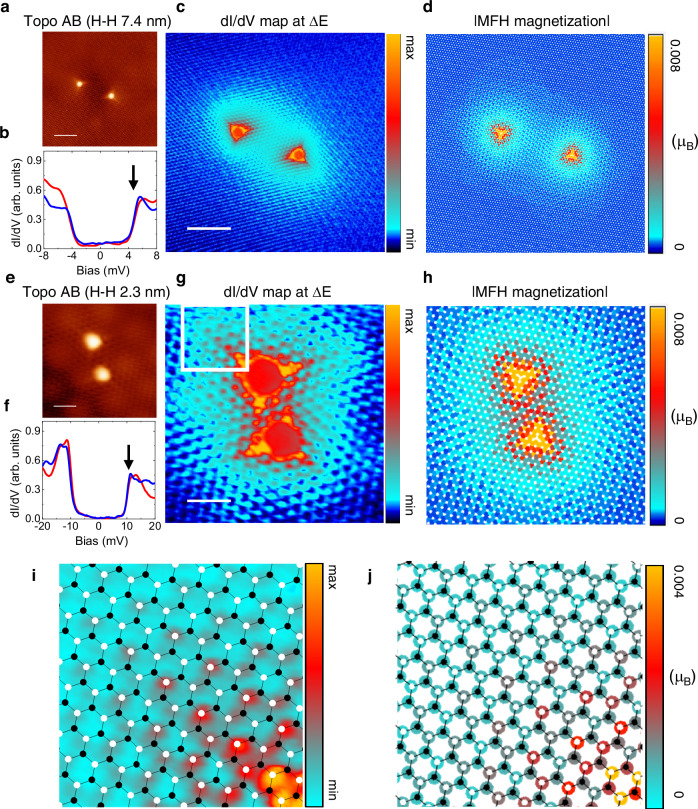
Spatially mapping spin excitations. **a** Topography image of an AB pair of H atoms at 7.4 nm distance (6 mV, 0.1 nA, scalebar: 5 nm). **b** *dI/dV* spectra measured on each of the H atoms in (**a**) (12 mV, 0.1 nA). The red (blue) curve has been measured on the left (right) atom. **c** *dI/dV* map at the excitation energy (6.3 mV, 0.1 nA, V_rms_ = 0.5 mV, scalebar: 5 nm). **d** Magnetization modulus of the antiferromagnetic ground state corresponding to experimental configuration shown in (**a**) calculated by MFH. **e** Topography image of an AB pair of H atoms at 2.2 nm distance (98 mV, 0.07 nA, scalebar: 1.5 nm). **f** *dI/dV* spectra measured on each of the H atoms in **e** (30 mV, 0.1 nA, scalebar: 1.5 nm). The red (blue) curve has been measured on the top (bottom) atom. **g** *dI/dV* map at the spin excitation energy (12 mV). **h** Magnetization modulus of the antiferromagnetic ground state corresponding to the experimental configuration shown in (**f**) calculated by MFH. **i** Zoom of the *dI/dV* map in (**g**) (white inset) with a superimposed graphene lattice showing sublattice polarization. **j** Zoom (2.3 × 2.3 nm2) of the magnetization in (**h**) a superimposed graphene lattice showing sublattice polarization.

Visualization of *dI/dV* maps at the characteristic spin-excitation energies of selected configurations provides deeper insight into the nature of these coupled states. As shown in Fig. 3, this approach enables spatially resolved mapping of spin excitations, directly probing the spatial extent and coherence of the magnetically coupled states.

In the first example, (Fig. 3a–d) we present an AB pair, placed at 7.4 nm distance, antiferromagnetically coupled with a measured excitation of ΔE = 5 meV. As anticipated by the unusually long-range coupling, the *dI/dV* map at this excitation energy shows a prominently bright region, surrounding the H atoms, indicating that we are able to induce the singlet to triplet transition even several nm away from the adsorption sites, unveiling the non-local nature of the induced magnetic moment. The amplitude of the *dI/dV* map relates to the spin spectral weight^26^. Our calculations of the spin excitation spectrum based on the random phase approximation (RPA), (see Supplementary Fig. 6), show that spin spectral weight map is very similar to the atomic magnetization map obtained in the mean field approximation. Therefore, we can also interpret the measured *dI/dV* maps (Fig. 3c) as a proxy for the local magnetization and compare them with MFH calculated magnetization (Fig. 3d).

In a second example, (Fig. 3f–j) we show an AB pair at a closer distance (2.2 nm), that presents a spin excitation at ΔE = 10 meV. The conductance map performed at this energy is again very similar to the MFH local magnetization. The detailed analysis of the interference pattern created on graphene, in both theory and experiment, shows that magnetization, and thus spin excitations, are sublattice polarized around each H atom (see Fig. 3i, j). In other words, placing the tip on either sublattice enables spin transitions with true atomic-scale selectivity. Importantly, while coupling occurs over unusually long distances, we can still resolve spin excitations and therefore the magnetization at the atomic scale.

### Spin S = 1/2 trimers

Building on our understanding of the magnetic coupling in 2H atom configurations and exploiting ultralong-range interactions together with STM’s atomic-manipulation capabilities, we can now scale up this approach by using individual H atoms as S = 1/2 building blocks to assemble larger spin networks. In this next step, we investigate the coupling that emerges when 3H atoms chemisorb on graphene. The relevant space of states for S = 1/2 trimers is composed of a quartet (S = 3/2) and two doublets (S = 1/2) (see SI8). Based on the sublattice of the chemisorption site, trimers come in two flavors, AAA and ABB. The spin of their ground state is expected to be S = 3/2 and S = 1/2, respectively, on account of the interplay between sublattice and exchange sign discussed above, and also by applying Lieb’s theorem. Importantly, once the IETS of a given trimer is taken, it is possible to selectively remove one H, resulting in a dimer whose exchange energy can be measured.

**Fig. 4:**
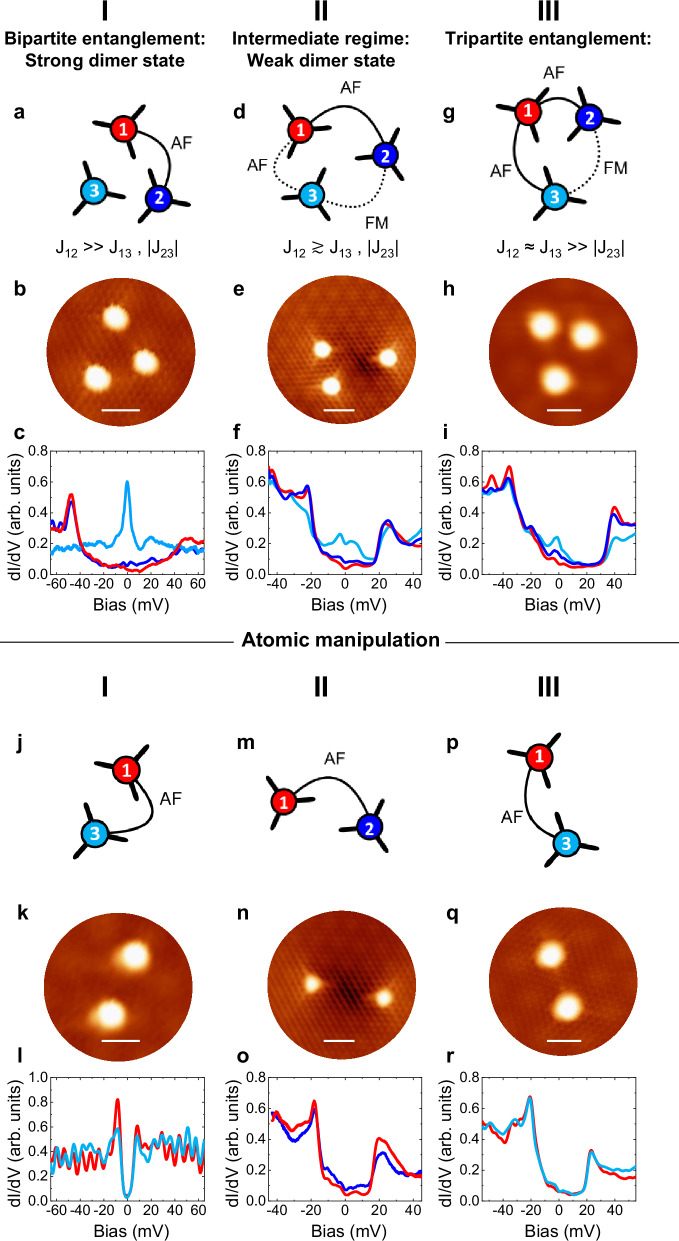
Three interacting S = 1/2 spins: ABB S = 1/2 configurations. **a** Schematics of bipartite entangled, dimerized ABB trimer (case I). **b** STM image of such ABB configuration (10 mV, 0.1 nA). **c** *dI/dV* spectra measured on each of the H atoms in (**b**) (60 mV, 0.1 nA). **d** Schematics of a weakly dimerized ABB trimer (case II). **e** STM image of such ABB configuration (4 mV, 0.05 nA). **f** *dI/dV* spectra measured on each of the H atoms in (**e**) (50 mV, 0.1 nA). **g** Schematics of a tripartite entangled ABB trimer dominated by antiferromagnetic interactions (case III). **h** STM image of such ABB configuration (400 mV, 0.05 nA). **i** *dI/dV* spectra measured on each of the H atoms in **h** (60 mV, 0.1 nA). **j** Schematics for case I after atomic-scale manipulation. **k** STM image of the configuration **b**, after atomic scale manipulation. **l** *dI/dV* spectra measured on each of the H atoms in (**k**) (60 mV, 0.1 nA). **m** Schematics for case II after atomic-scale manipulation. **n**, STM image of the configuration **e**, after atomic scale manipulation. **o** *dI/dV* spectra measured on each of the H atoms in (**n**) (50 mV, 0.1 nA). **p** Schematics for case III after atomic-scale manipulation. **q** STM image of the configuration (**h**), after atomic scale manipulation. **r** *dI/dV* spectra measured on each of the H atoms in (**q**) (60 mV, 0.1 nA). The red, dark blue, and light blue curves in (**c, f, i, l, o**, and **r**), correspond to the H sites indicated with the same color in the schemes in (**a, d, g, j, m,** and **p**), respectively. FM and AFM abbreviations denote ferromagnetic and antiferromagnetic coupling, respectively. The scale bar is 2 nm.

In the present work, we focus on ABB trimers with an S = 1/2 ground state to investigate how distinct coupling regimes, set by the ratios of pairwise spin interactions, are reflected in their IETS spectra (for completeness, Supplementary Fig. 7 presents data for the AAA, S = 3/2 system). Figure 4 presents IETS data for three ABB trimers at similar interatomic separations, and how their spectra evolve when one hydrogen atom is removed. In all cases, the trimer spectra (Figs. 4c, 4f, 4i) differ significantly from those of the corresponding dimers (Fig. 4l, 4o, 4r), clearly demonstrating the presence of exchange interactions between the removed hydrogen atom and the remaining pair. Furthermore, the inelastic excitations observed in the remaining dimer spectra reinforce the existence of exchange coupling between these two hydrogens, as previously discussed. Thus, the data consistently indicates a system with the possibility of having three exchange-coupled spins.

Depending on the relative strength of the three pairwise exchange interactions in an ABB trimer, two distinct limiting regimes can be identified. In practice, the coupling regime is inferred directly from the experimental *dI/dV* line shapes, from the energy, and relative intensity of inelastic steps measured at the different atomic sites. In the first regime, characterized by one spin on the B sublattice being weakly coupled and the remaining AB pair strongly coupled (Fig. 4a–c), the IETS spectrum of the weakly coupled spin significantly deviates from that of the strongly coupled dimer. It lacks spin excitations and instead resembles the spectrum of an isolated hydrogen atom.

In contrast, the strongly coupled pair displays a spectrum similar to those shown in Figs. 1 and 3, featuring a single excitation step. This regime corresponds to bipartite entanglement. The assignment of the coupling hierarchy in this case follows directly from the presence or absence of inelastic steps (Fig. 4a, c).

In the opposite regime, the antiferromagnetic exchange couplings are comparable in magnitude and collective spin excitations dominate. Consequently, we expect similar spectra across all three atomic sites. This behavior is clearly illustrated in Fig. 4g–i, where spectra on all three sites share a common inelastic feature at around 34 meV, indicative of a collective spin excitation and thereby of tripartite entanglement. This collective spin excitation has similar intensity on all three sites.

Furthermore, ABB trimers provide an excellent framework to investigate intermediate coupling scenarios between these two extremes. Such an intermediate situation is illustrated by the trimer shown in Fig. 4d–f, where a strongly coupled AB dimer exhibits a common excitation at 20 meV (data points 1 and 2), also visible in the spectrum of the third, less coupled B spin (data point 3), albeit with significantly reduced intensity. We classify this scenario as a weak dimer state (Fig. 4d).

In the first limiting case described earlier (weak coupling of the third spin), the strongly coupled antiferromagnetic dimer achieves maximal bipartite entanglement. As coupling to the third spin strengthens, entanglement monogamy dictates that bipartite entanglement within the dimer is progressively reduced, giving rise to genuine tripartite entanglement^42^. Due to the clear distinction in IETS signatures between these two extremes, we propose that spectral features observed in these systems could serve as measurable indicators of tripartite entanglement.

One could also expect that some trimers feature two clear inelastic steps, corresponding to transitions from the S = 1/2 ground state to the excited states with either S = 1/2 and S = 3/2 (see SI7-8). However, in our experiments, we have not clearly identified that situation. We attribute this to the fact that the visibility of two steps is possible whenever the splitting of the two inelastic steps is larger than the IETS resolution (see Supplementary Figs. 1 and 8). In Supplementary Fig. 9 we plot a simulation of how the energy difference between excited states depends on the exchange couplings. We find that a significant fraction of the phase diagram does not comply with the visibility criteria.

In summary, our work demonstrates that the controlled hydrogenation of graphene down to the single-atom level unlocks robust, tunable magnetic moments with long-range and sublattice-dependent spin coupling. By assembling atomic-scale spin clusters—dimers, trimers, quartets, quintets, and hexamers (Supplementary Fig. 10), and imaging their spin excitations with atomic precision, we establish a powerful platform for engineering designer spin Hamiltonians on demand. Looking ahead, combining this exquisite control with tunable carrier density promises to further tailor spin interactions in situ, while integrating superconducting proximity effects could open pathways to investigate exotic quantum phases at the confluence of magnetism and superconductivity^43,44^. Together, these advances herald new directions in quantum materials design and quantum simulation at the atomic scale.

## Methods

### Sample preparation and experimental details

Graphene was grown epitaxially by thermal decomposition of SiC^45,46^, and atomic H was randomly deposited on the upmost graphene layer by cracking a molecular beam of H_2_. Most chemisorbed H atoms (~95%) form non-magnetic dimers even for very dilute concentrations^47,48^, thus, after H deposition, we use STM manipulation to build the desired magnetic H arrangements^28^. The magnetic configurations were fabricated using a two-step process: first, H atoms were gathered with the STM tip by rapidly scanning the surface at low bias voltages (5–100 mV) and high tunneling currents (5–10 nA), second, the collected H atoms were then deposited onto a chosen graphene region using negative sample voltage pulses (up to −9 V). To achieve the intended dimer or trimer configuration, excess H atoms are subsequently removed one by one. This is done by approaching the STM tip to within 1–2 Å of the adsorption site, which allows selective desorption of individual H atoms with essentially 100% efficiency. As a result, the final positioning accuracy of the H-atom arrangements is atomic-scale, despite the minor uncertainty in the initial deposition step. This combination of coarse, repeatable deposition followed by deterministic single-atom removal enables the precise and reproducible construction of the targeted magnetic configurations with nanometer-scale precision. The procedure is illustrated in SI Fig. 10 and has been described in detail previously, with sequential images, in Refs. ^28,41^.

The measurements were performed on a low-temperature STM operating at 3 K. To facilitate spectroscopy measurements, we used Pb superconducting tips prepared, after H manipulation, by indentation on Pb nanoislands previously deposited on graphene. The presented *dI/dV* spectra were numerically deconvoluted to remove the effect of the superconducting tip unless indicated otherwise. By performing *dI/dV* curves on our configurations of H atoms, we can access the magnetic excitations of our system thanks to Inelastic Electron Tunneling Spectroscopy (IETS). The signature of spin excitations in IETS is an increase in the *dI/dV* spectra, symmetric at both bias polarities^19^. These excitation energies correspond to the exchange coupling between spins and thus provide a quantification of the strength of the magnetic coupling. The STM data was acquired and processed using the software WSxM^49^.

### Theory methods

We describe hydrogenated graphene using a Hubbard model in the mean field approximation,

1 $$H=t{\sum}_{ < {ij} > }{\sum}_{\sigma }{a}_{i\sigma }^{{{\dagger}} }{a}_{j\sigma }+U{\sum}_{i}{\sum}_{\sigma }\left[\left\langle {a}_{i\bar{\sigma }}^{{{\dagger}} }{a}_{i\bar{\sigma }}\right\rangle {a}_{i\sigma }^{{{\dagger}} }{a}_{i\sigma }-\left\langle {a}_{i\bar{\sigma }}^{{{\dagger}} }{a}_{i\bar{\sigma }}\right\rangle \left\langle {a}_{i\sigma }^{{{\dagger}} }{a}_{i\sigma }\right\rangle \right]$$

where *t* is the nearest-neighbor hopping integral, *U* is the on-site (screened) Coulomb repulsion parameter, σ=↑,↓ denotes the spin direction, *i,j* are atomic site indices and *a*^*†*^_iσ_ (*a*_iσ_) creates (annihilates) an electron state with spin σ at site *i*. The angular brackets denote expected values in the mean-field configuration. As a chemisorbed hydrogen atom removes one p orbital and one electron from the honeycomb lattice, it can be modeled as a vacancy^50^, keeping the ratio of electrons per carbon site equal to one (half-filling). We use a simulation cell with periodic boundary conditions with up to *N* = 16000 sites. We adopt the value of 2.7 eV for the nearest-neighbor hopping integral *t*, and the Hubbard parameter *U* = |*t*| for all the calculations shown. The excitation energy for a pair of chemisorbed H atoms is defined as the total energy difference between the ferromagnetic and antiferromagnetic mean-field configurations. Each magnetic configuration, characterized by the site-dependent populations of the states associated with the two spin directions, is determined self-consistently. The populations for up and down spin states at each site are allowed to vary freely, under the following constraints: in the antiferromagnetic configuration the total number of electrons for both spin directions, *N*_↑_ and *N*_↓_, is the same (*N*_↑ _= *N*_↓_); in the ferromagnetic configuration, *N*_↑ _= *N*_↓ _+ 1; for both configurations the total number of electrons is fixed at half-filling, *N*_↑ _+ *N*_↓ _= *N*.

## Supplementary information

Supplementary Information

Transparent Peer Review file

## Data Availability

The data that support these findings are available from the corresponding authors upon request.
