# Supplementary material for: Ultra-long-range spin coupling in graphene revealed by atomically resolved spin excitations
Source: Nat Commun. 2026 May 19;17:6586. doi: 10.1038/s41467-026-72624-0 (PMC13381549; doi:10.1038/s41467-026-72624-0)
Supplement: Supplementary file 1 — Supplementary Information [file 41467_2026_72624_MOESM1_ESM.pdf]

Supplementary Information. Ultra-long-range spin coupling in graphene revealed by atomically resolved spin excitations

Authors: Beatriz Viña-Bausá[1]*, António Tavares Costa[2,3], Joao Henriques[2,4], Eva Cortés-del Río[5], Roberto Carrasco[1], Pierre Mallet[6], Jean-Yves Veuillen[6], Joaquín Fernández-Rossier[2]*, Iván Brihuega[1,7]*

Affiliations:

[1]*Departamento de Física de la Materia Condensada, Universidad Autónoma de Madrid; E-28049 Madrid, Spain*

[2]*International Iberian Nanotechnology Laboratory (INL); Avenida Mestre José Veiga, 4715-310 Braga, Portugal*

[3]*Physics Center of Minho and Porto Universities (CF-UM-UP), Universidade do Minho; Campus de Gualtar, 4710-057 Braga, Portugal*

[4]*Universidade de Santiago de Compostela, Santiago de Compostela, Spain.*

[5]*Department of Physics, University of Hamburg; D-20355 Hamburg, Germany.*

[6]*Université Grenoble Alpes, Grenoble, F-38400 France and CNRS, Institut Néel; Grenoble, F-38042 France*

[7]*Condensed Matter Physics Center (IFIMAC) and Instituto Nicolás Cabrera (INC), Universidad Autónoma de Madrid; E-28049 Madrid, Spain*

** Corresponding authors*

email: beatriz.vina@uam.es; joaquin.fernandez-rossier@inl.int; ivan.brihuega@uam.es

Supplementary Information

1. Extensive study of magnetic coupling in AB and AA pairs

2. Spin excitation at higher temperatures

3. Distance dependence magnetic coupling and comparison with other systems

4. Orientational degree of freedom in AB pairs

5. Mapping spin excitations and extension of coupled states

6. AAA configuration (S=3/2 ground state) and manipulation to ferromagnetic dimer

7. Heisenberg description of trimers

8. Analytical solution of Heisenberg trimer model

9. Building coupled structures with more than 3H atoms

SI1. Extensive study of magnetic coupling in AB and AA pairs

AB	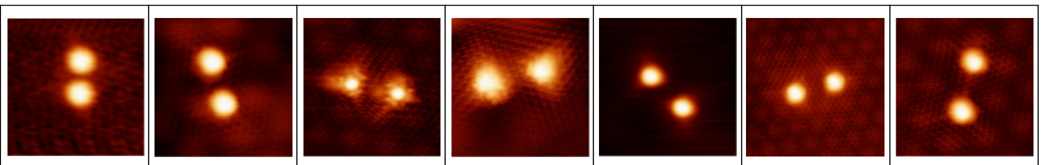						
$^{dI}/_{dV}$	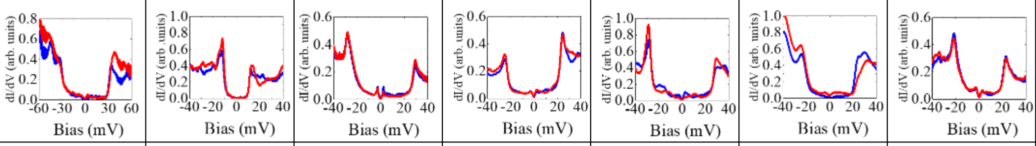						
Deconv	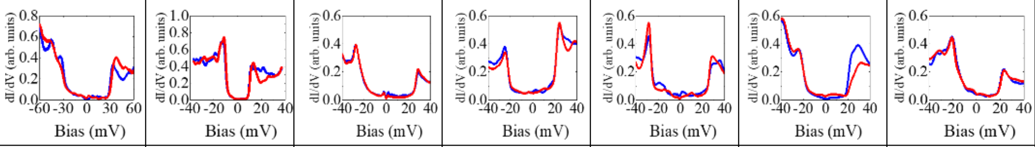						
$^{dI^2}/_{dV^2}$	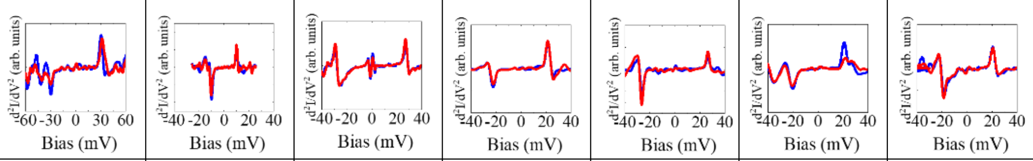						
H-H distance	1.7 nm	2.2 nm	2.3 nm	2.5 nm	2.9 nm	2.9 nm	3.1 nm
ΔE	31 meV	10 meV	27 meV	22 meV	27 meV	21 meV	20 meV

AB	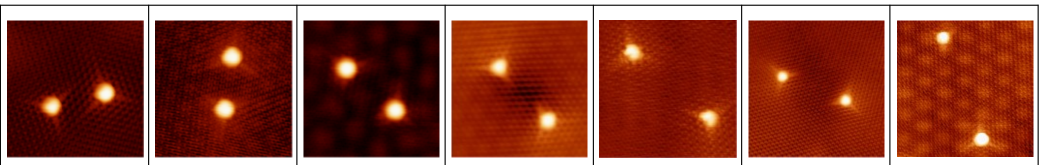						
$^{dI}/_{dV}$	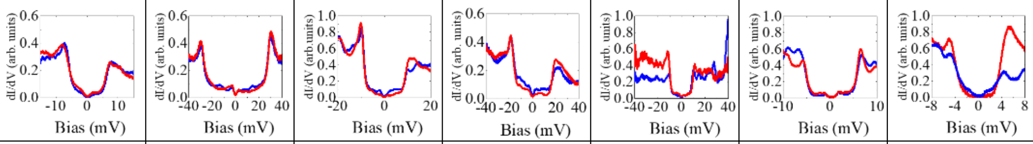						
Deconv	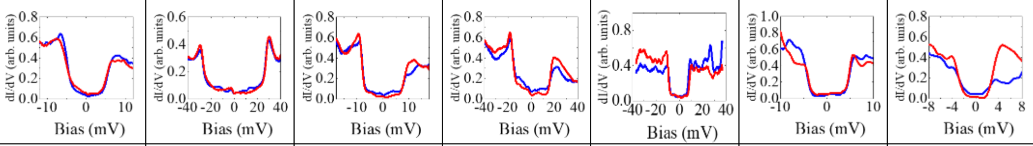						
$^{dI^2}/_{dV^2}$	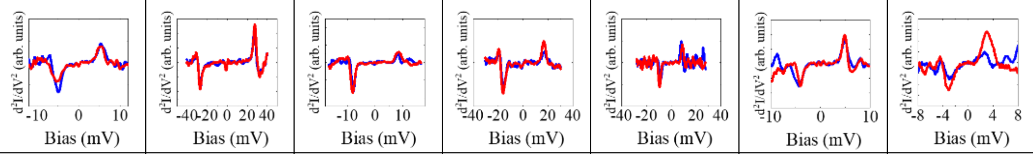						
H-H distance	3.1 nm	3.6 nm	4.0 nm	4.4 nm	5.9 nm	7.4 nm	10.7 nm
ΔE	5 meV	27 meV	8 meV	17 meV	9 meV	5 meV	3 meV

Supplementary Table 1: Spin excitations in configurations of H atoms in opposite graphene sublattices. In the first row we present the STM topography images of the configurations. In the second and third rows we show raw spectra measured with a superconducting tip and its numerical deconvolution respectively. The fourth row shows the numerical, second order derivative. The fifth and sixth row contain the distance between H atoms and the magnitude of the spin excitation, respectively.

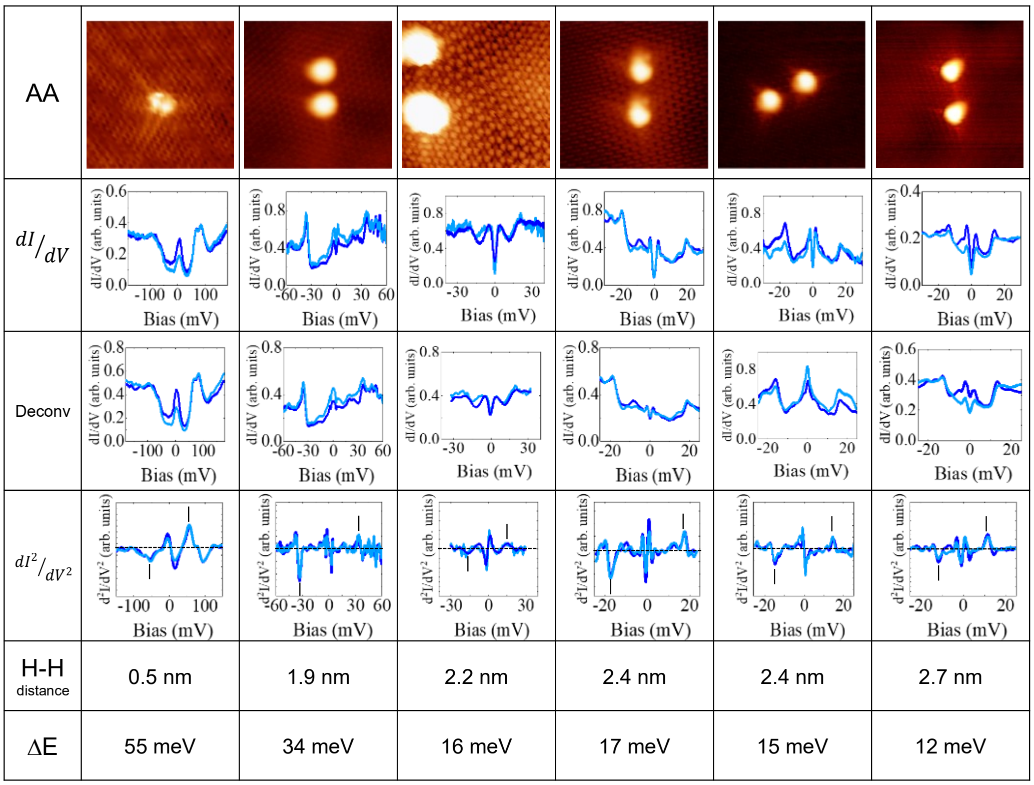

H-H distance	0.5 nm	1.9 nm	2.2 nm	2.4 nm	2.4 nm	2.7 nm
ΔE	55 meV	34 meV	16 meV	17 meV	15 meV	12 meV

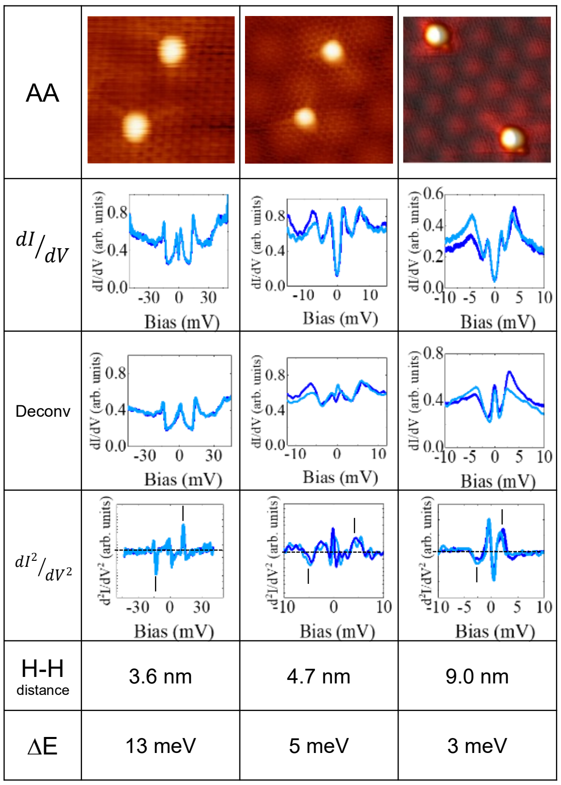

H-H distance	3.6 nm	4.7 nm	9.0 nm
ΔE	13 meV	5 meV	3 meV

Supplementary Table 2: Spin excitations in configurations of H atoms in the same graphene sublattices. In the first row we present the STM topography images of the configurations. In the second and third rows we show raw spectra measured with a superconducting tip and its numerical deconvolution respectively. The fourth row shows the numerical, second order derivative. The fifth and sixth row contain the distance between H atoms and the magnitude of the spin excitation, respectively. Black lines have been included to identify the spin excitations in the second order derivative.

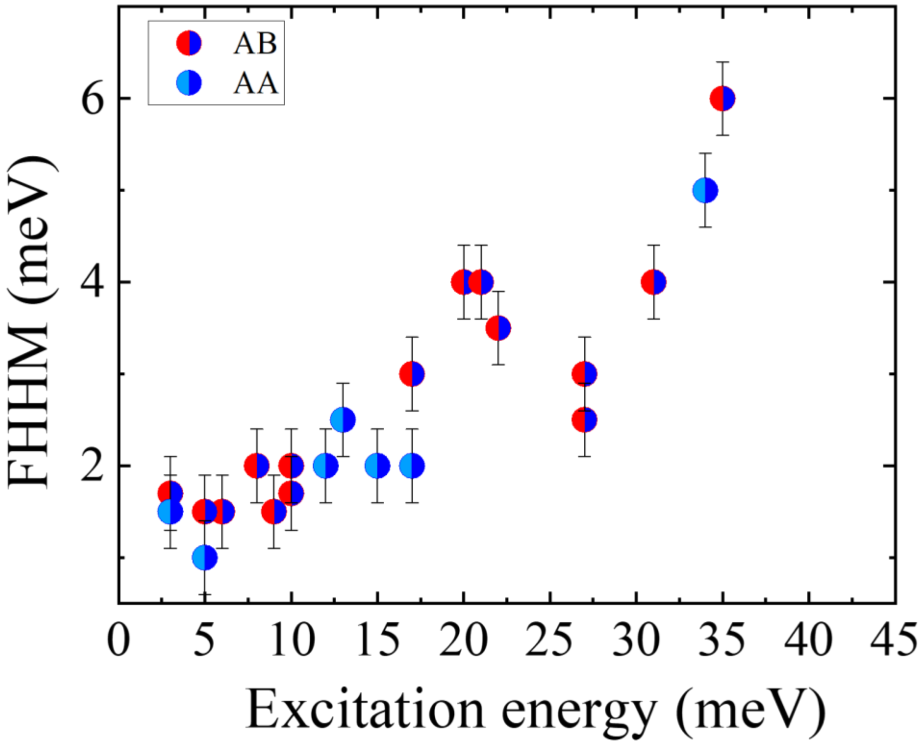

Supplementary Fig. 1: IETS energy resolution. Full width at half maximum (FWHM) of the inelastic spin excitations obtained from the numerical second derivative as a function of the excitation energy. Red-blue (blue-blue) dots correspond to AB (AA) pairs. Error bars are included in the FWHM to account for positive-negative bias asymmetries.

SI2. Spin excitations at higher temperatures

AB pair from Supplementary Table 1 (top panel, column 3)

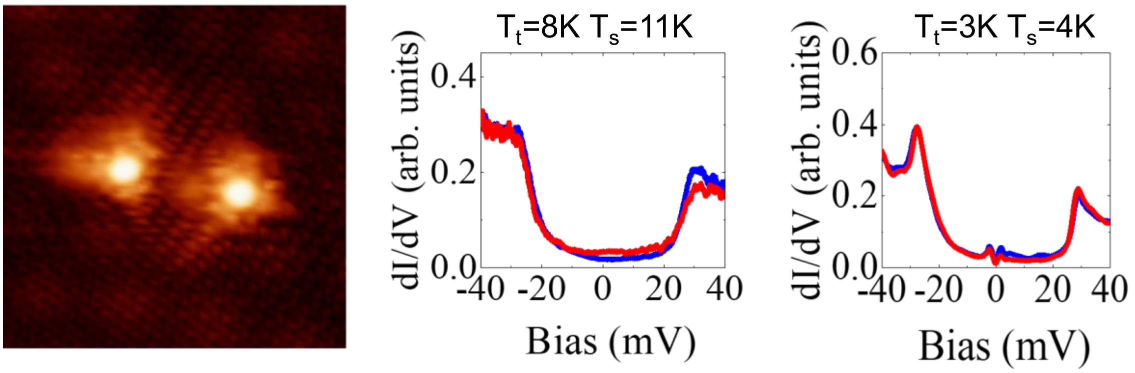

AB pair from Supplementary Table 1 (top panel, column 4)

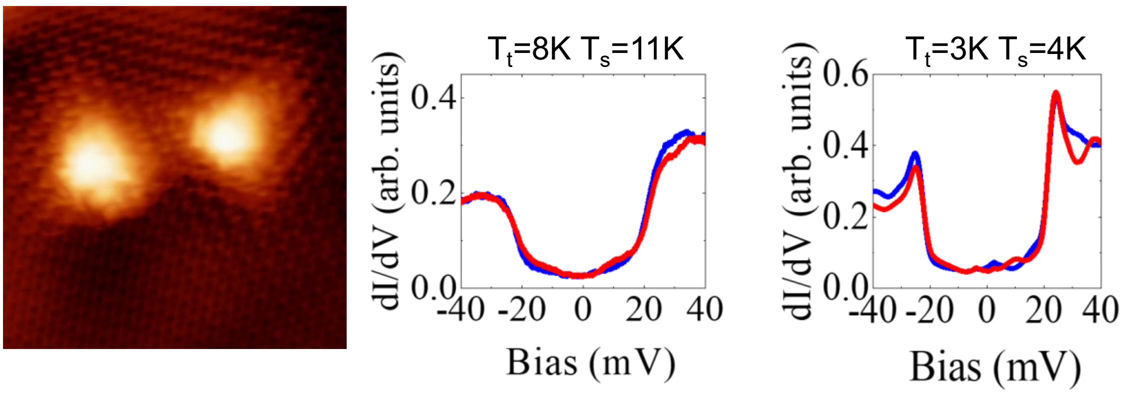

Supplementary Fig. 2: Spin excitations in AB pairs measured with the sample (T_s) at 11 K and the tip (T_t) at 8 K. (Top): AB from chart S1, corresponding to the configuration in the third column of the top panel. The red (blue) curve has been measured on the left (right) H atom. (Bottom): AB from chart S1, corresponding to the configuration in the fourth column of the top panel. The red (blue) curve has been measured on the left (right) H atom.

SI3. Distance dependence of magnetic coupling and comparison with other systems

a

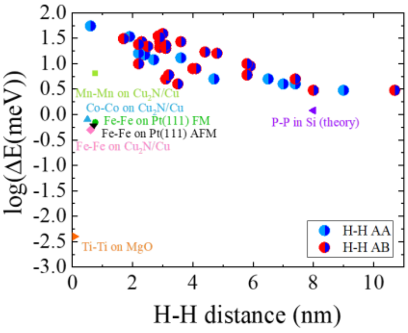

b

R(Å)	J (mev)	$Log_{10}(J)$	|J| R	System	Reference
90	-5	0.70	450	H on graphene (AA) furthest	This work
107	3	0.48	321	H on graphene (AB) furthest	This work
5	-55	1.74	275	H on graphene (AA) closest	This work
17	31	1.49	527	H on graphene (AB) closest	This work
7.6	6.2	0.79	47.12	Mn-Mn on Cu2N/Cu	18
7.6	-0.7	-0.16	5.32	Fe-Fe on Cu2N/Cu(100)	36
5	-0.8	-0.01	4	Co-Co on Cu2N/Cu(100)	37
7	-0.6	-0.22	4.2	Fe-Fe on Pt(111)	38
6	0.5	-0.30	3	Fe-Fe on Pt(111)	38
0.64	0.004	-2.4	0.00256	Ti-Ti on MgO	39
80	1.2	0.08	96	P-P in Silicon	40

c

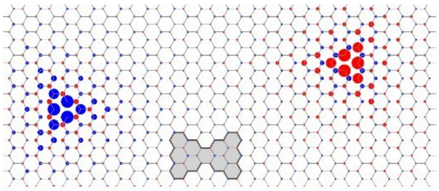

Supplementary Fig. 3: Distance dependence of magnetic coupling and comparison with other systems. a, Experimental exchange coupling in logarithmic scale as a function of distance between magnetic moments in hydrogenated graphene (this work) and in different compounds from refs. *18, 36-40*. **b,** Summary of the exchange energies and the interaction distance magnitude in this work and in different compounds from refs. *18, 36-40*. **c,** Comparison of lengths scales with nanographenes: Atomic magnetization map of the AB pair of H atoms of Fig 1.c of the main text, with singlet-triplet splitting of 17 meV, superimposed with the molecular structure of Clar's goblet, with a measured[23] singlet-triplet splitting of 23 meV. Red and blue colors indicate opposite spin orientation.

SI4. Orientational degree of freedom in AB pairs

The relative orientation of the induced magnetic moments is governed by the anisotropic nature of the spin generated by hydrogen chemisorption on the graphene honeycomb lattice. As observed in STM images and reported in ref. 28, a single hydrogen adatom induces a threefold-symmetric magnetic pattern with a $\sqrt{3}x\sqrt{3}$ periodicity, reflecting the underlying lattice symmetry. The relative orientation between two induced moments is therefore determined by the alignment of these threefold patterns, which can be directly visualized experimentally and in calculations.

Three limiting configurations can be identified (see Fig. SI4): (i) H atoms aligned along the graphene armchair direction in a head-to-head arrangement, (ii) armchair alignment in a back-to-back configuration, and (iii) alignment along the zigzag direction. In practice, H pairs can adopt relative orientations that lie anywhere between these extreme cases. This orientational degree of freedom, in addition to the H–H distance, determines the strength of the exchange interaction and thus the spin excitation energies observed in dimers and trimers.

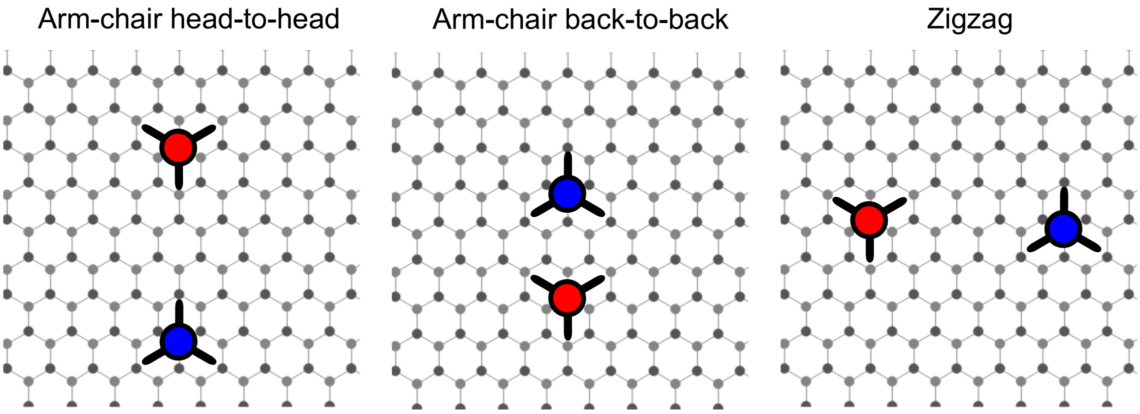

Supplementary Fig. 4. Limiting orientational configurations of H pairs.

Thus, the apparent lack of a simple correlation between H–H distance and excitation energy for antiferromagnetically coupled AB pairs, both in experiments and calculations can be attributed to the additional orientational degree of freedom associated with the anisotropic induced magnetic moments. In Fig.SI5 we provide calculations of the distance dependence exchange energy at a fixed orientation between AB pairs.

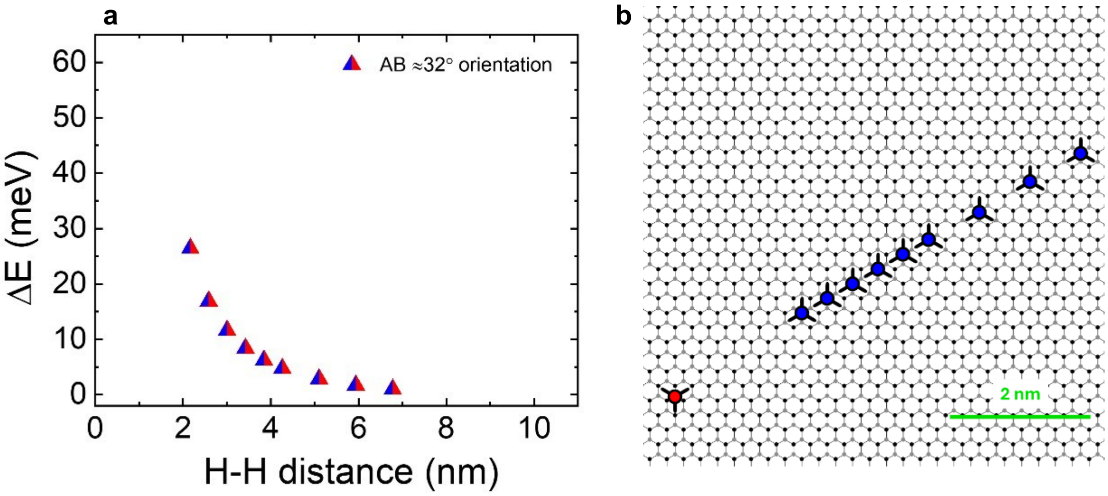

Supplementary Fig. 5. Monotonous decay of the exchange coupling for AB pairs at a fixed orientation. a, Calculated exchange coupling as a function of the relative distance. **b**, Atomic configurations corresponding to the calculations. Position of atom A (red) is fixed, and atom B (blue) is positioned at different distances.

SI5. Mapping spin excitations and extension of coupled states

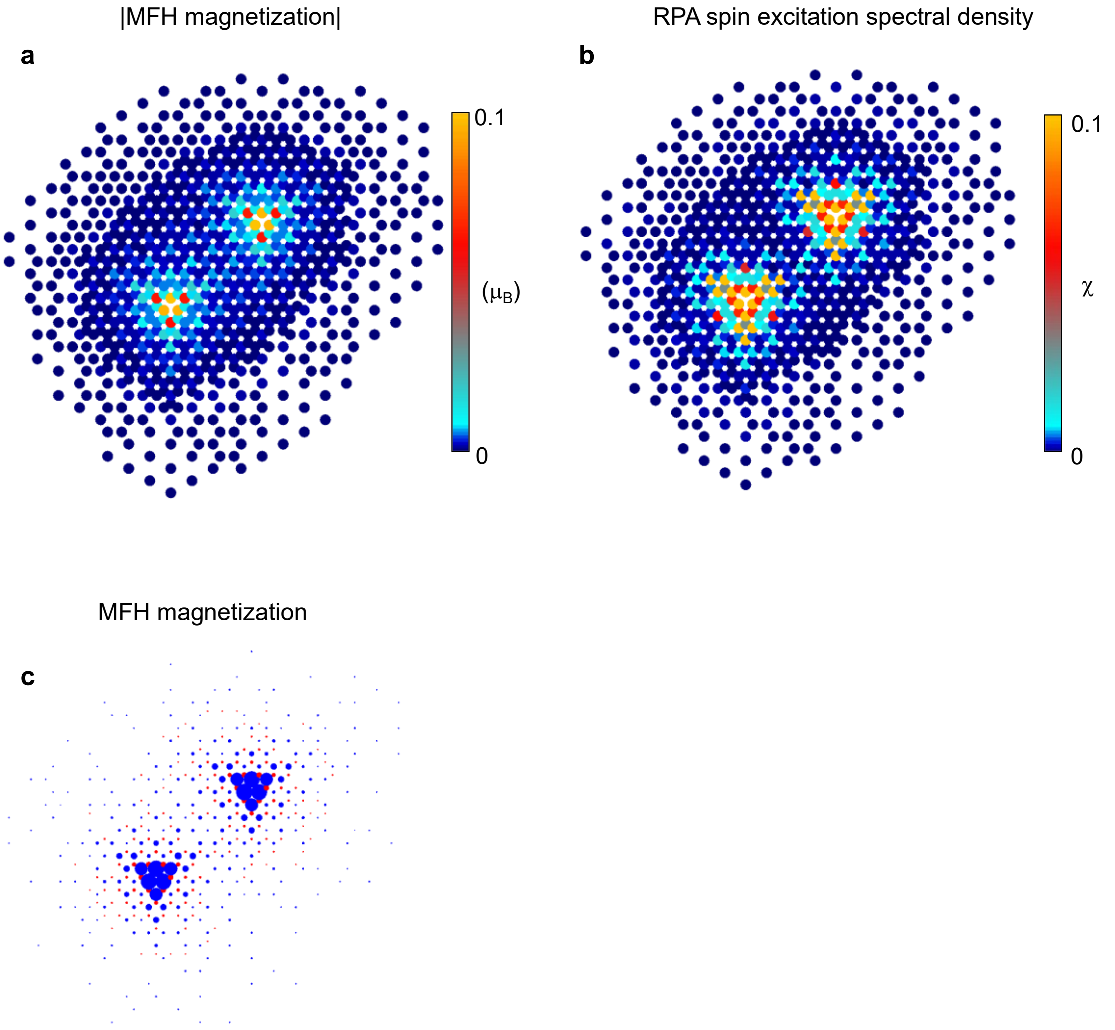

Supplementary Fig. 6: AA MFH equilibrium magnetization map and random phase approximation (RPA) spectral weight of an AA configuration. a, Absolute value of the MFH magnetization of an AA configuration at 2.4 nm. **b,** RPA calculated spectral density corresponding to the triplet to singlet excitation of the same configuration in **a**. The color scale has been adjusted to render a better comparison with the magnetization. **c,** Magnetization showing the ferromagnetic ground state of the configuration, dot size is proportional to the magnetization magnitude and blue and red indicate opposite spin orientations.

SI6. AAA configuration (S=3/2 ground state) and manipulation to ferromagnetic dimer

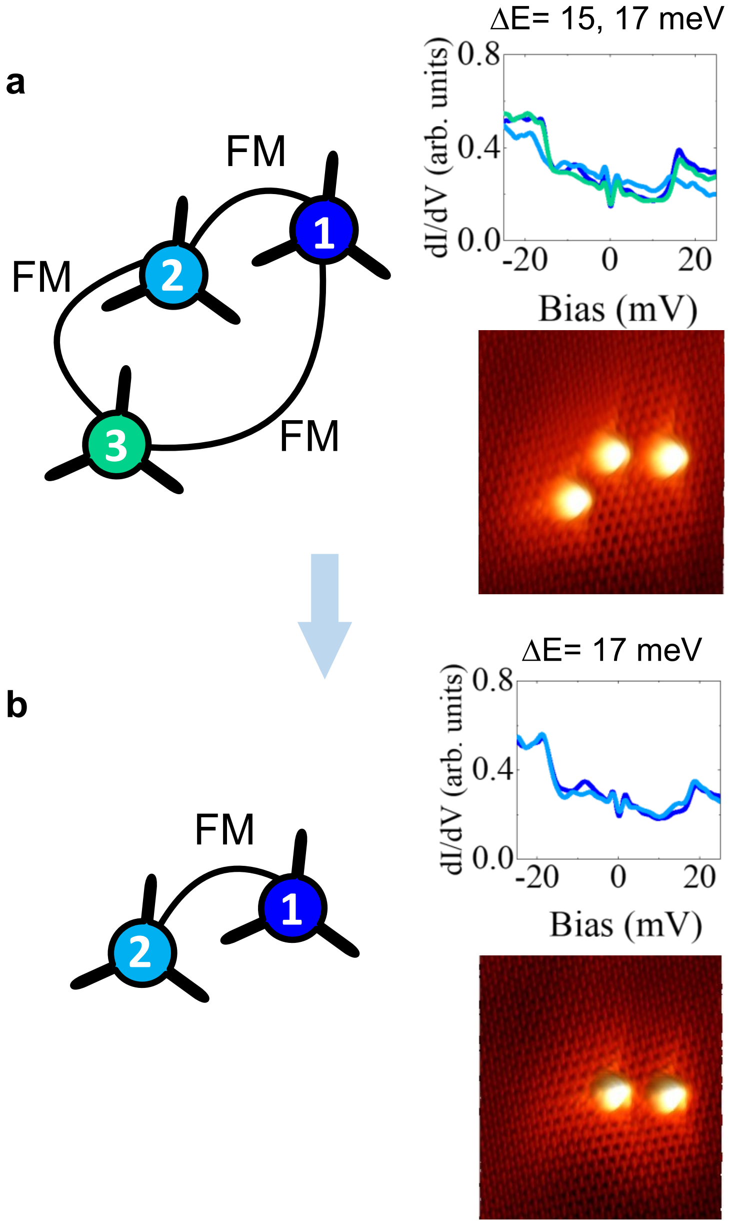

Supplementary Fig. 7: AAA trimer. a, *dI/dV* spectra, STM image and coupling diagram of an AAA trimer configuration. **b,** Atomic scale manipulation of the configuration in **a**. *dI/dV* spectra, STM image and coupling diagram after the removal of H3. The dark blue, light blue and green curves were measured on the H sites indicated with the same color in the scheme. FM indicates ferromagnetic coupling.

SI7. Heisenberg model description of trimers

We solved the Heisenberg model using pairwise exchange couplings estimated from experiments as input parameters to model the trimers. Figure S8 presents the workflow and provides a comparative summary for the experimental configuration II shown in Fig. 4 of the main text. The calculated excitation energies from the Heisenberg model qualitatively agree with the experimental observations, highlighting its effectiveness in capturing key aspects of the system's magnetic behavior.

Nevertheless, the Heisenberg model does not fully describe the complete behavior observed in trimers. In particular, it does not reproduce the lower-bias features associated with the addition peaks originating from localized states of the hydrogen atoms (particularly pronounced at site 3). Additionally, the conductance step heights linked to the amplitude of spin excitations at each site are not precisely captured. Despite these quantitative differences, the relative increase in conductance at transition energies is qualitatively comparable between theory and experiment (see panel d).

The magnetic moments are delocalized over the graphene lattice, a factor expected to significantly shape the spatial distribution of excitations. Thus, reducing the system to a simplified three-site model may overlook important nuances. Importantly, the Heisenberg model identifies certain transitions close in energy, which might remain unresolved experimentally due to broader linewidths originating primarily from relaxation mechanisms involving graphene continuum states (see SI Fig. 1).

Furthermore, the introduction of a third hydrogen atom could potentially introduce screening or enhancement effects in coupling, significantly influencing spin spatial distribution and magnetic transitions. These considerations motivate the development of more comprehensive models, capable of capturing beyond-pairwise exchange interactions, fermionic characteristics, and graphene-induced anisotropy. Exploring these aspects in greater detail will be the focus of future research.

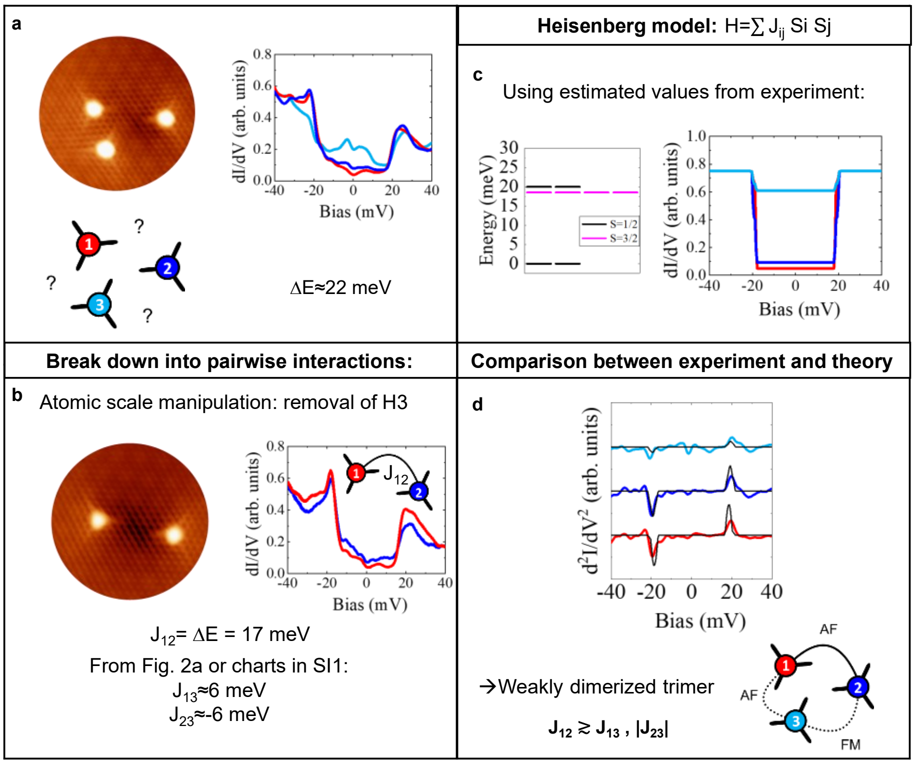

Supplementary Fig. 8. Experimental measurements and modeling of trimers. a, ABB trimer configuration with *dI/dV* spectra on the 3 sites showing a clear spin excitation. The red, dark blue

and light blue curves were measured on the H sites indicated with the same color in the scheme below. **b,** Atomic-scale manipulation of the configuration in **a**. By removing H3 we can access the experimental exchange coupling between spins 1 and 2 (J_{12}). The red and dark blue curves were measured on the H sites indicated with the same color in the scheme. The remaining couplings (J_{13}, J_{23}) are estimated from the distance dependence plot and other experiments to try to fit best the measured excitation spectrum of the trimer. **c,** Simulated energy levels and *dI/dV* with Heisenberg model and tunneling current up to second order with the exchange couplings from **b**. The red, dark blue and light blue curves correspond to the sites indicated with the same color in the scheme in **a**. **d,** Comparison of the experimental data (colors) and Heisenberg model (black lines) using the second derivative of the *dI/dV*. The red, dark blue and light blue curves were measured on the H sites indicated with the same color in the scheme below.FM and AFM indicates ferromagnetic and antiferromagnetic coupling, respectively.

SI8. Analytical solution of Heisenberg trimer model

Let us consider the following Hamiltonian which describes three spins, interacting via linear exchange which is allowed to be different for each pair of spins:

$$H = J\left(\boldsymbol{S}_1 \cdot \boldsymbol{S}_2 + \alpha \boldsymbol{S}_2 \cdot \boldsymbol{S}_3 + \beta \boldsymbol{S}_1 \cdot \boldsymbol{S}_3\right)$$

where J > 0 is the exchange interaction between spins 1 and 2; Jα and Jβ are the exchange interactions between spins 2 and 3, and spins 1 and 3, respectively. Alternatively, we can write this Hamiltonian as

$$\frac{H}{J} = S_1^z S_2^z + \alpha S_2^z S_3^z + \beta S_1^z S_3^z + \frac{S_1^+ S_2^- + S_1^- S_2^+}{2} + \alpha \frac{S_2^+ S_3^- + S_2^- S_3^+}{2} + \beta \frac{S_1^+ S_3^- + S_1^- S_3^+}{2}$$

where we split the Hamiltonian into an Ising like, and a flip-flop contribution. The spectrum of this Hamiltonian is made of 8 states, which are arranged into two doublets (S = 1/2) and one quartet (S = 3/2). Since the Hamiltonian commutes with S^2 and S^z, we know that the states with $S^z = \pm 3/2$ are given by $|3/2, +3/2\rangle = |\uparrow\uparrow\uparrow\rangle$ and $|3/2, -3/2\rangle = |\downarrow\downarrow\downarrow\rangle$. These are eigenstates of the Hamiltonian, whose energy is entirely determined by the Ising part of the Hamiltonian:

$$H|3/2, +3/2\rangle = \frac{J}{4}(1 + \alpha + \beta)|3/2, +3/2\rangle$$
$$H|3/2, -3/2\rangle = \frac{J}{4}(1 + \alpha + \beta)|3/2, -3/2\rangle$$

To find the eigenergies of the = 1/2 states with $S^z = +1/2$ we note that these can be written in terms of the S^2 eigenstates:

$$|+\rangle = \frac{1}{\sqrt{3}}\left(|\downarrow\uparrow\uparrow\rangle + \omega_+|\uparrow\downarrow\uparrow\rangle + \omega_+^2|\uparrow\uparrow\downarrow\rangle\right)$$
$$|-\rangle = \frac{1}{\sqrt{3}}\left(|\downarrow\uparrow\uparrow\rangle + \omega_-|\uparrow\downarrow\uparrow\rangle + \omega_-^2|\uparrow\uparrow\downarrow\rangle\right).$$

Now, our goal is to express the Hamiltonian in this basis, and then diagonalize it. Computing the Hamiltonian matrix elements between the $|+\rangle$ and the $|-\rangle$ states, we find

$$\langle +|H|+\rangle = -\frac{1}{4}(1 + \alpha + \beta)$$
$$\langle -|H|+\rangle = \frac{1}{4}\left(-1 + i\sqrt{3} + 2\alpha - \left(1 + i\sqrt{3}\right)\beta\right)$$
$$\langle +|H|-\rangle = \frac{1}{4}\left(-1 - i\sqrt{3} + 2\alpha - \left(1 - i\sqrt{3}\right)\beta\right)$$
$$\langle -|H|-\rangle = -\frac{1}{4}(1 + \alpha + \beta)$$

Which gives the matrix representation

$$H = -\frac{J}{4}\begin{pmatrix} 1 + \alpha + \beta & 1 - i\sqrt{3} - 2\alpha + \left(1 + i\sqrt{3}\right)\beta \\ 1 + i\sqrt{3} - 2\alpha + \left(1 - i\sqrt{3}\right)\beta & 1 + \alpha + \beta \end{pmatrix}$$

whose eigenvalues read

$$E_{\pm} = -\frac{J}{4}\left(1+\alpha+\beta \pm 2\sqrt{1+\alpha^2+\beta\left(\beta-1\right)-\alpha(1+\beta)}\right)$$

Thus, we find that the Heisenberg Hamiltonian for the trimer with inhomogeneous couplings gives three manifolds.

In the limit where one of the spins is weakly coupled to the other two, i.e. α, β ≪ 1, we find the following approximate expressions for the energy levels:

$$\begin{aligned} E(S=3/2) &= \frac{J}{4}+\frac{J}{4}\left(\alpha+\beta\right) \\ E(S=1/2) &\approx -\frac{3J}{4}+\mathcal{O}(\alpha\beta) \\ E(S=1/2) &\approx \frac{J}{4}-\frac{J}{2}\left(\alpha+\beta\right)+\mathcal{O}(\alpha\beta) \end{aligned}$$

Thus, for the case where one of the spins is weakly coupled to the other one, the ground state is a S = 1/2 doublet, and the first excited state can be either S = 1/2 or S = 3/2 depending on α and β (which can be positive or negative). The absolute energy difference between the two excited manifolds, Δ, reads

$$\Delta = \frac{3J}{4}\left|(\alpha+\beta)\right|$$

Consider now the limit where two of the exchanges are similar, and have the same sign. Without loss of generality, we take α ≈ 1, and take β as the free parameter. Then:

$$\begin{aligned} E(S=3/2) &= \frac{J}{4}\left(2+\beta\right) \\ E(S=1/2) &= -\frac{J}{4}\left(2+\beta+2\sqrt{\left(\beta-1\right)^2}\right) \\ E(S=1/2) &= -\frac{J}{4}\left(2+\beta-2\sqrt{\left(\beta-1\right)^2}\right) \end{aligned}$$

Since, by definition, -1 < β < 1, then β - 1 < 0. Therefore:

$$\begin{aligned} E(S=3/2) &= \frac{J}{2}+\frac{J}{4}\beta \\ E(S=1/2) &= -J+\frac{J}{4}\beta \\ E(S=1/2) &= -\frac{3J}{4}\beta \end{aligned}$$

where we once again find the ground state to be an S = 1/2 doublet. The first excited state is also a doublet, and the quartet appears as the highest energy state.

Alternatively, we now consider α ≈ −1, and take β as the free parameter. Then:

$$E(S = 3/2) = \frac{J}{4}\beta$$
$$E(S = 1/2) \approx -\frac{\sqrt{3}}{2}J - \frac{J}{4}\beta + \mathcal{O}(\beta^2)$$
$$E(S = 1/2) \approx +\frac{\sqrt{3}}{2}J - \frac{J}{4}\beta + \mathcal{O}(\beta^2)$$

As before we find the ground state to be an S = 1/2 doublet, only this time the first excited state is the S = 3/2 quartet, and the other S = 1/2 doublet appears as the highest energy manifold.

In Fig. S9 we plot the energy difference between the two excited states of a trimer as a function of the coupling parameters α and β and we locate the experimental configurations of Fig. 4 of the main text.

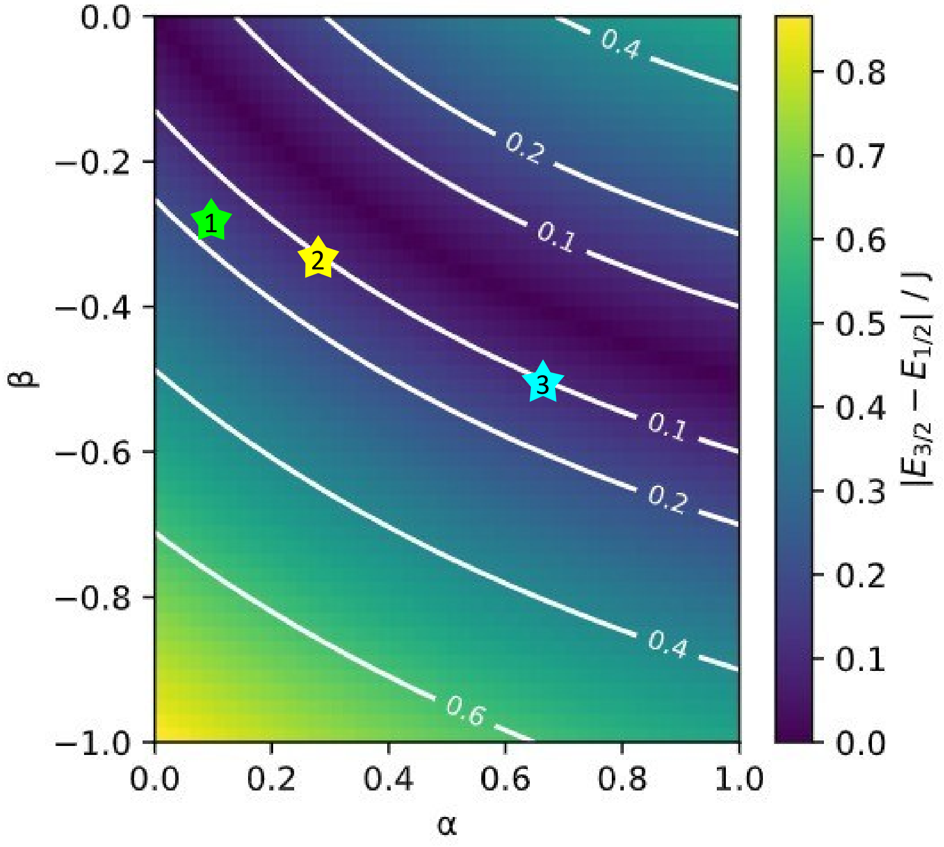

Supplementary Fig. 9. Excitation spectra for ABB trimers as a function of the ratios of pairwise exchange couplings α and β. Splitting between the trimer excited states for fixed J (J12) and for J23= β*J and J13= α*J. Stars correspond to the experiments I, II and III shown in Fig. 4 of the main text.

SI9. Building coupled structures with more than 3H atoms

In Fig. S10, we present an experiment beginning with a configuration of 8H atoms. The data reveal that the induced magnetic moments are collectively coupled, as evidenced by the appearance of multiple inelastic excitation features (highlighted by dashed lines in Fig. S10b). Further support for this coupling comes from sequential removal of individual H atoms, which leads to significant changes in the spectra (see Fig. S10c–h).

By examining the evolution *dI/dV* curves during each atomic-scale manipulation, we are able to track site-specific changes. For instance, on site 1 (H1), one prominent spin excitation shifts from nearly 30 meV to 12 meV. Across all manipulation steps, multiple spin excitations are consistently observed, marked with tentative dashed lines in the bias-symmetric features in the spectra and summarized in Fig. S10j.

These observations demonstrate the tunability of this spin system, highlighting its potential for scalable architectures based on engineered magnetic interactions at the atomic level.

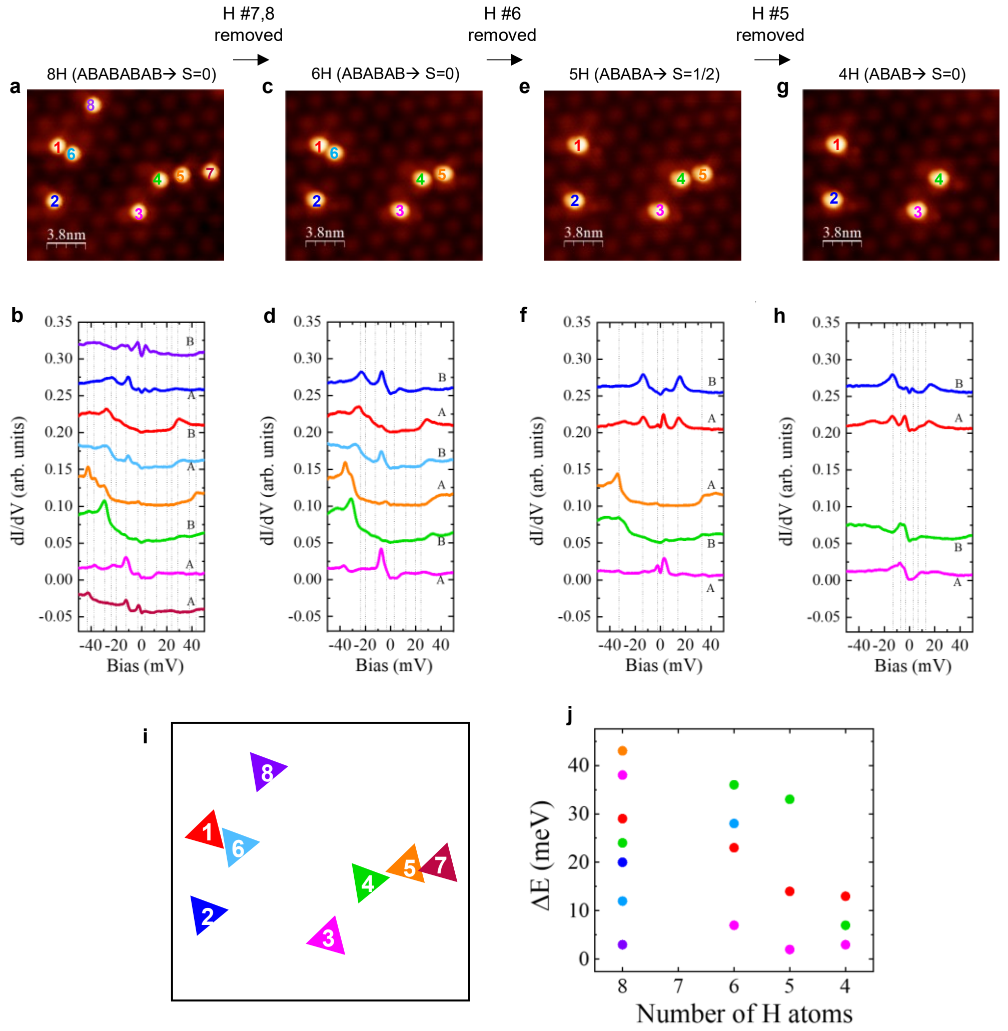

Supplementary Fig. 10. Building coupled structures with more than 3H atoms, visualization of spin excitations and manipulation. a, Configuration with 8H atoms and S=0. **b,** Spin excitations probed on each H site. Up to 7 spin excitations can be identified. **c,** Configuration with 6H atoms and S=0. **d,** Spin excitations probed on each H site. Up to 4 spin excitations can be identified. **e,** Configuration with 5H atoms and S=1/2. **f,** Spin excitations probed on each H site. Up to 3 spin excitations can be identified. **g,** Configuration with 4H atoms and S=0. **h,** Spin excitations probed on each H site. Up to 3 spin excitations can be identified. **i,** Schematics of the initial configuration with 8H atoms. **j,** Collective spin excitations in each situation with a different

total number of H atoms. The color of the *dI/dV* spectra in **b, d, f** and **g** correspond to the H sites indicated in **a, c, e** and **g** with the same colors.
