# Supplementary material for: Ultra-long-range spin coupling in graphene revealed by atomically resolved spin excitations
Source: Nat Commun. 2026 May 19;17:6586. doi: 10.1038/s41467-026-72624-0 (PMC13381549; doi:10.1038/s41467-026-72624-0)
Supplement: Supplementary file 2 — Transparent Peer Review file [file 41467_2026_72624_MOESM2_ESM.pdf]

nature portfolio

Peer Review File

Ultra-long-range spin coupling in graphene revealed by atomically resolved spin excitations

Corresponding Author: Professor Iván Brihuega

This file contains all reviewer reports in order by version, followed by all author rebuttals in order by version

Parts of this Peer Review File have been redacted as indicated to maintain the confidentiality of unpublished data.

Version 0:

Reviewer comments:

Reviewer #1

(Remarks to the Author)
In this manuscript, the authors investigate the creation and control of long-range magnetic exchange interactions in spin-½ pairs and trimers achieved through hydrogen atom chemisorption on graphene. The study effectively integrates scanning tunneling microscopy/spectroscopy (STM/STS) experiments with large-scale theoretical modeling, including mean-field Hubbard and random phase approximation calculations. The authors report substantial exchange couplings of up to 3 meV at notably long distances exceeding 10 nm, accompanied by atomic-scale control over the ferromagnetic or antiferromagnetic nature of the interaction. The results are substantiated by extensive datasets, and the manuscript is clearly structured. These findings establish the atomic hydrogen chemisorbed in graphene as a platform for spin-based quantum technologies. Before recommending publication, however, I do have several comments to this work:

1.How repeatable is the hydrogen chemisorption process in terms of positioning accuracy during measurements?

2.What is the exact number of graphene layers used in this study? In regions exhibiting a moiré superlattice, could the twist angle between graphene layers influence the observed spin behavior or the conclusions?

3.Additional inelastic electron tunneling signatures are occasionally observed near the Fermi level for the 2H atoms adsorbed in the AB configuration, such as at the H–H distance of 3.1 nm listed in Table 1. What is the physical origin of these states?

4.For AB pairs with H–H separations larger than 10 nm, are the authors able to find low-energy spectral features similar to those characteristic of monomeric H adsorbates?

5.The authors identify three tuning parameters—distance, sublattice arrangement, and orientation relative to the crystallographic axis—that govern the strength and sign of exchange interactions. How is the relative orientation of the anisotropically induced magnetic moments determined? Can the exchange interactions in dimer and trimer configurations be considered deterministic or random?

6.Additionally, for antiferromagnetically coupled AB pairs, the spin excitation energies in calculation do not appear to correlate directly with H–H distance. Even at same distance (e.g., 2.1 nm and 6.9 nm), the spin excitation energies differ. Given the importance of this relationship, additional discussion elaborating on the underlying factors would be valuable.

7.How is the consistency of the STM tip verified following hydrogen atom manipulation? The little differences in STM images between trimer and dimer configurations raise the question of tip stability. Moreover, what is the origin of the additional spectral peaks near the inelastic tunneling peaks in Fig. 4i, and should they be regarded as physically meaningful?

Minor Suggestions:
8.Tables 1 and 2 should be organized in a systematic order (e.g., by increasing H-H distance) to improve readability.

9.All scale bars in Fig. 4 and the Supplementary Information should be visible.

Reviewer #2

(Remarks to the Author)
The manuscript by Vina-Bausa et al. reports on the investigation of magnetic interactions of localized spin-1/2 centers realized as chemisorbed hydrogen atoms on graphene. Scanning tunneling microscopy at low temperatures is implemented to observe the low-voltage differential conductance signatures (inelastic electron tunneling spectroscopy) giving insights into the spin states of the system. Interactions within dimers are investigated revealing pronounced exchange couplings at distances as large as 10 nm. In trimers and clusters with higher number of spins magnetic collective spin excitations are studied. Overall I find the paper interesting and appealing to the general physics community and in particular to those studying electron correlations in solids. Both experimental and theoretical data are convincing. The presentation of the manuscript is very clear and of high quality. I am fully persuaded that Nature Communications is the right journal for these findings. In particular with the current manuscript the authors establish a handy system which allows for the on-demand realization of complex spin systems. Thus, providing an important toolkit going beyond the already existing efforts. Thus I suggest publication with minor corrections.

Minor questions:
I wonder about the actual degree of control within the system. Is it possible to place the spin centers at particular graphene cites on demand or to move attached hydrogen atoms along the graphene lattice to particular positions? Or is it more a question of chance to get a desired nanostructure. In the supplementary material part the hydrogen removal process is shown, but the overall control of the actual position of the hydrogen atoms is undiscussed. Would it be possible to saturate the surface with hydrogen and to remove the hydrogen overshoot by the STM tip leaving the desired structure behind? I would happy to see the authors' ideas along these lines.

In the manuscript the authors the data on trimers (Fig. 4). I would be interested to se a more detailed discussion of the line shapes observed in the STS spectra presented in c,f,i. How the couplings are assigned departing from the experimental results?

Reviewer #3

(Remarks to the Author)
The authors present a detailed study of spin correlations between H atoms adsorbed on graphene. This is accomplished by means of inelastic electon tunneling spectroscopy and STS, from which the spin-triplet gap is extracted and converted into an effective exchange coupling between the spins. The control of H atoms on graphene is remarkable and the entire study is backed up by mean-field Hubbard calculations, which provides a good agreement and explanation with the results. While the results and experimental techniques are rather impressive, it is not clear to me if there are any big surprises in here - perhaps the magnitude of exchange splitting in the H-dimers, but is is not so clear what one would expect. Nevertheless, he paper is well written and comprises a nice and coherent story. It will be interesting for a broad readership and I thus recommend publication. A few comments below:

It would be useful if the results were compared with the singlet-triplet splitting of a bare H-dimer. Perhaps as a function of distance obtained by some numerical method. This would likely be a different order of magnitude but still a useful comparison.

The authors mention several exchange mechanisms at play. Direct, kinetic and RKKY. Could the authors try to quantify the contribution of these. Right now, the statement is a bit trivial because is merely states the possible mechanisms.

Version 1:

Reviewer comments:

Reviewer #1

(Remarks to the Author)
The authors have conducted a comprehensive and high-quality revision of the manuscript in response to the previous review comments, leading to a marked improvement in the manuscript quality. This work features notable innovation, systematic design and significant scientific importance, fully meeting the publication criteria of Nature Communications, and is therefore recommended for immediate acceptance.
By virtue of elaborate experimental design and in-depth theoretical analysis, the authors report the first observation of ultra-long-range spin coupling exceeding 10 nm with a coupling strength of 3 meV in graphene, breaking the spatial and energy scale limits of traditional spin systems. Combining STM atomic manipulation and IETS spectroscopic characterization, the authors realize the precise fabrication of spin dimers and trimers, achieve atomic-resolution imaging of spin excitations, and further reveal the sublattice-dependent ferromagnetic/antiferromagnetic coupling modulation as well as collective spin excitations and quantum entanglement characteristics in trimers. These findings provide a novel material platform and experimental approach for quantum magnetism and spin-based quantum computing, holding great scientific value and application potential.
The revised manuscript is excellent in supplementary data, result interpretation and figure optimization. The extensive additional experimental data and simulation results further verify the reliability of the core conclusions; the analysis of spin coupling mechanisms and spectroscopic features is more in-depth and thorough; the figures are clear and intuitive, and highly consistent with the textual discussion.

In summary, this work is characterized by novel research content, comprehensive and solid experimental data, and sufficient and persuasive argumentation. The authors have successfully completed the manuscript revision with no further revisions required. It is recommended for immediate publication in Nature Communications.

Reviewer #2

(Remarks to the Author)
The manuscript by Vina-Bausa et al. reports on the investigation of magnetic interactions of localized spin-1/2 centers realized as chemisorbed hydrogen atoms on graphene. Scanning tunneling microscopy at low temperatures is implemented to observe the low-voltage differential conductance signatures (inelastic electron tunneling spectroscopy) giving insights into the spin states of the system.

Overall I find the paper interesting and appealing to the general physics community. Both experimental and theoretical data are convincing. The authors fully addressed the questions raised by the referees and improved the manuscript accordingly. I am confident that Nature Communications is the right journal for these findings. Thus I support the acceptance of the manuscript.

Reviewer #3

(Remarks to the Author)
The manuscript may be published in its current form

Open Access This Peer Review File is licensed under a Creative Commons Attribution 4.0 International License, which permits use, sharing, adaptation, distribution and reproduction in any medium or format, as long as you give appropriate credit to the original author(s) and the source, provide a link to the Creative Commons license, and indicate if changes were made.
In cases where reviewers are anonymous, credit should be given to 'Anonymous Referee' and the source.
The images or other third party material in this Peer Review File are included in the article’s Creative Commons license, unless indicated otherwise in a credit line to the material. If material is not included in the article’s Creative Commons license and your intended use is not permitted by statutory regulation or exceeds the permitted use, you will need to obtain permission directly from the copyright holder.
To view a copy of this license, visit https://creativecommons.org/licenses/by/4.0/

Reviewer #1 (Remarks to the Author):

In this manuscript, the authors investigate the creation and control of long-range magnetic exchange interactions in spin-½ pairs and trimers achieved through hydrogen atom chemisorption on graphene. The study effectively integrates scanning tunneling microscopy/spectroscopy (STM/STS) experiments with large-scale theoretical modeling, including mean-field Hubbard and random phase approximation calculations. The authors report substantial exchange couplings of up to 3 meV at notably long distances exceeding 10 nm, accompanied by atomic-scale control over the ferromagnetic or antiferromagnetic nature of the interaction. The results are substantiated by extensive datasets, and the manuscript is clearly structured. These findings establish the atomic hydrogen chemisorbed in graphene as a platform for spin-based quantum technologies. Before recommending publication, however, I do have several comments to this work:

We would first like to thank the reviewer for their careful reading of the manuscript and positive comments on our work.

1.How repeatable is the hydrogen chemisorption process in terms of positioning accuracy during measurements?

The hydrogen chemisorption process is repeatable with nanometer-scale positioning accuracy. The magnetic configurations are fabricated using a two-step procedure.
First, H atoms are collected with the STM tip by rapidly scanning the surface at low bias voltages (5–100 mV) and high tunneling currents (5–10 nA). Second, the collected H atoms are deposited onto a selected graphene region by applying negative sample-voltage pulses (up to −9 V). This deposition step reproducibly delivers a variable number of H atoms positioned randomly to a targeted region in the order of 30 nm^2.
To achieve the intended dimer or trimer configuration excess H atoms are subsequently removed one by one. This is done by approaching the STM tip to within 1–2 Å of the adsorption site, which allows selective desorption of individual H atoms with essentially 100% efficiency. As a result, the final positioning accuracy of the H-atom arrangements is atomic-scale, despite the minor uncertainty in the initial deposition step.
This combination of coarse, repeatable deposition followed by deterministic single-atom removal enables the precise and reproducible construction of the targeted magnetic configurations with nanometer scale precision. The procedure is illustrated in SI Fig. 10 and has been described in detail previously, with sequential images, in *Science*, **352**, 437-441 (2016) (Fig. 4) and *Nano Letters* ***25***, 11554-11561 (2025) (Fig. 2).
Following the referee's comment, we have modified the "**Methods"** section in the manuscript to provide more details that facilitate a clear understanding of this procedure.

2.What is the exact number of graphene layers used in this study? In regions exhibiting a moiré superlattice, could the twist angle between graphene layers influence the observed spin behavior or the conclusions?

In our work, to ensure the charge neutrality of the surface graphene layer, a "thick" (5-7 graphene layers) multilayer graphene sample was grown. We performed experiments on two independently prepared samples, epitaxially grown on two different SiC substrates, and obtained consistent results across both systems.
For this material system, it is well established that the upmost graphene layer is essentially electronically decoupled and behaves as free-standing graphene due to rotational disorder for twisting angles larger than 10°. Importantly, previous work (*Science* **352**, 437–441 (2016)) showed that, provided graphene remains charge neutral, the influence of the underlying layers on

the hydrogen-induced magnetic moments is minimal. In this regime, hydrogen chemisorption generates magnetic moments almost exclusively on the top graphene layer.

For the data presented here, acquired in regions with large twist angles, the moiré superlattice is therefore expected to have only a residual influence on the induced magnetic moments and their relative couplings and, consequently, on the measured spin excitation spectra.

We note, however, that exploring hydrogen-induced magnetism in graphene systems with small twist angles, in particular close to the magic angle, where flat bands and strong correlations emerge, would be highly interesting. In such regimes, the interaction between magnetic impurities and correlated electronic states could lead to qualitatively new phenomena, but this lies beyond the scope of the present study.

3.Additional inelastic electron tunneling signatures are occasionally observed near the Fermi level for the 2H atoms adsorbed in the AB configuration, such as at the H–H distance of 3.1 nm listed in Table 1. What is the physical origin of these states?

We thank the referee for pointing this out. In the data of the AB pair at 3.1 nm with the excitation energy of 20 meV of Table 1 we had mistakenly included the second derivative of the raw *IV* curve instead of the deconvoluted data, therefore that produces an intense feature at the Fermi level originating from the superconducting gap of the tip (see Fig. 1). We have now corrected this mistake in the manuscript and included the second derivative of the deconvoluted spectra.

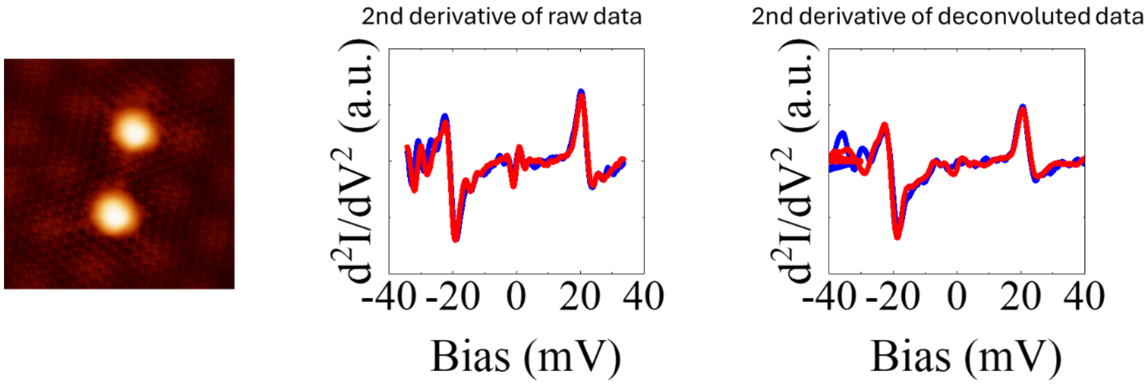

Figure 1: STM image (left) of an AB configuration and d^2I/dV^2 spectra from the raw data (middle) and the deconvoluted data (right).

However, a reduced amount of AB pairs do show weak peaks at the Fermi level, approximately an order of magnitude smaller in intensity than the spin excitations. We have carefully looked at the data and believe that these features can arise from the presence of impurities even if far away. Specifically, for the configuration at 2.3 nm distance and 27 meV excitation energy in the data at 4K, which shows the most prominent peak at E_F, we have found that there was a third H atom at >8 nm that is likely to be responsible for the low energy features in the *dI/dV* spectra, present even after the deconvolution of the superconducting tip (see Figure 2). In this same configuration, measured at higher temperature and after removing the additional H, we observe that the inelastic features remain at the same energy (see SI Fig. 2), indicating that the extra H had essentially no effect in the coupling between the close AB pair.

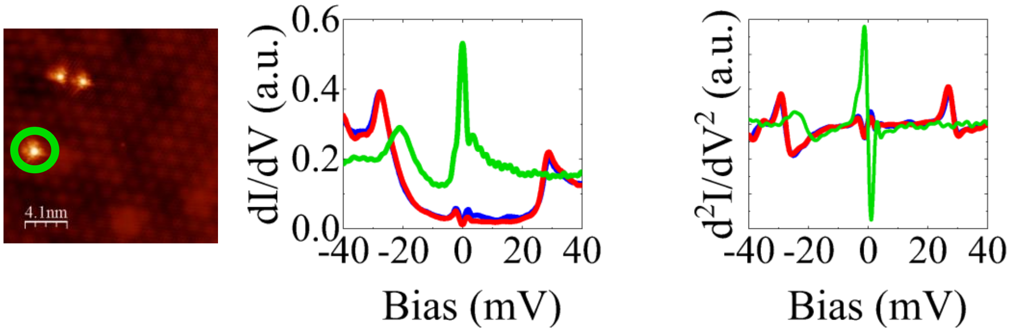

Figure 2: STM image (left) of an AB configuration with a single H at 8 nm, deconvoluted *dI/dV* (middle) and d^2I/dV^2 (right) spectra on the AB pair and single H.

We therefore conclude that these subtle low-energy features, which may arise from the nonlocal character of electronic states in graphene, do not affect the dominant spectral signatures associated with spin excitations. Nevertheless, because these features are reproducible, experimentally robust, and not attributable to noise, likely reflecting the local environment, we prefer not to select data arbitrarily, but instead to present them all.

4.For AB pairs with H–H separations larger than 10 nm, are the authors able to find low-energy spectral features similar to those characteristic of monomeric H adsorbates?

In short, when H atoms are separated more than 10 nm from any other H atom, we find the main characteristic of a monomeric H adsorbate i.e. the presence of spin split addition peaks with energy separations of the order of 2-15 meV, depending on the local environment.

However, we unfortunately do not have sufficiently clean AB data in the range of 10-20 nm that could reply to the fair question raised by the referee. We agree that the observation of spin-split peaks at the Fermi level, measured on each of the H atoms far away, and the absence of spin excitations, would truly reveal whether the coupling completely disappears beyond 10 nm. At present, we are unable to draw a definitive conclusion from our data, primarily due to the presence of other defects within a similar distance range, but this interesting remark surely deserves attention and needs to be studied in the future.

5.The authors identify three tuning parameters—distance, sublattice arrangement, and orientation relative to the crystallographic axis—that govern the strength and sign of exchange interactions. How is the relative orientation of the anisotropically induced magnetic moments determined? Can the exchange interactions in dimer and trimer configurations be considered deterministic or random?

6.Additionally, for antiferromagnetically coupled AB pairs, the spin excitation energies in calculation do not appear to correlate directly with H–H distance. Even at same distance (e.g., 2.1 nm and 6.9 nm), the spin excitation energies differ. Given the importance of this relationship, additional discussion elaborating on the underlying factors would be valuable.

We provide our answers to questions 5 and 6 together:

In short, the exchange interactions in dimer and trimer configurations are deterministic, i.e. 2 or 3 H atoms showing the same relative arrangement, will always present the same exchange interaction, with marginal contribution from the local environment.
However, the referee is correct by pointing out that H dimers with very similar separations show a very different magnitude of their magnetic couplings, both in experiments and calculations. This is a very robust observation related to the relative orientations between H atoms.

The relative orientation of the induced magnetic moments is governed by the anisotropic nature of the spin generated by hydrogen chemisorption on the graphene honeycomb lattice. As observed in STM images and reported in *Science*, **352**, 437-441 (2016), a single hydrogen adatom induces a threefold-symmetric magnetic pattern with a $\sqrt{3}x\sqrt{3}$ periodicity, reflecting the underlying lattice symmetry. The relative orientation between two induced moments is therefore determined by the alignment of these threefold patterns, which can be directly visualized experimentally and in calculations.

Three limiting configurations can be identified (see Fig. 3): (i) H atoms aligned along the graphene armchair direction in a head-to-head arrangement, (ii) armchair alignment in a back-to-back configuration, and (iii) alignment along the zigzag direction. In practice, H pairs can adopt relative orientations that lie anywhere between these extreme cases. This orientational degree of freedom, in addition to the H–H distance, determines the strength of the exchange interaction and thus the spin excitation energies observed in dimers and trimers.

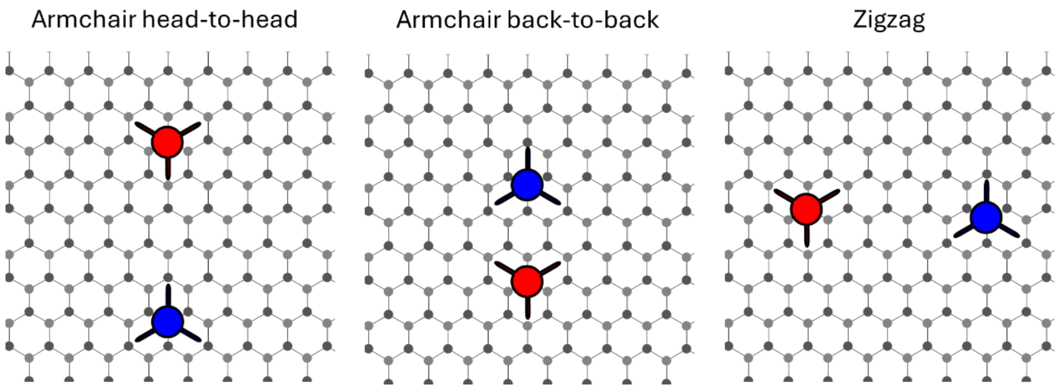

Figure 3: Limiting orientational configurations of H pairs.

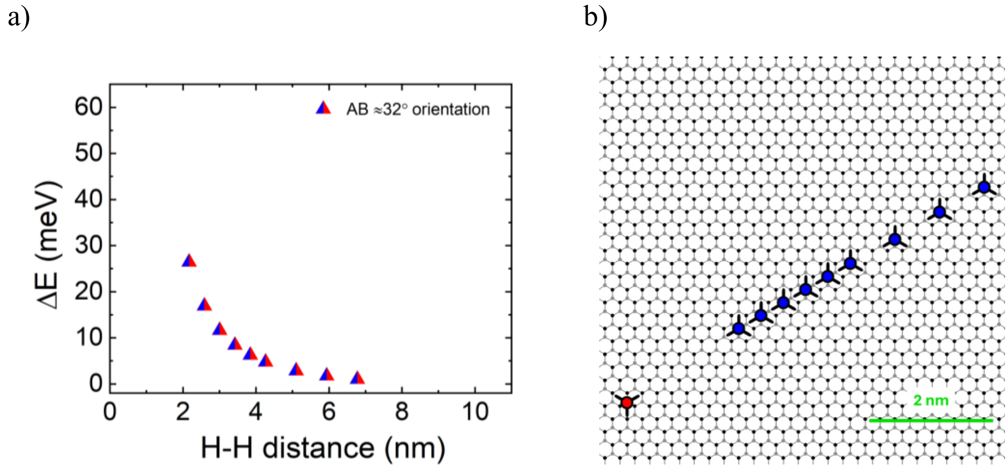

Figure 4: Monotonous decay of the exchange coupling for AB pairs at a fixed orientation. a) Calculated exchange coupling as a function of the relative distance. b) Atomic positions corresponding to the calculated configurations.

Thus, the apparent lack of a simple correlation between H–H distance and excitation energy for antiferromagnetically coupled AB pairs, can be attributed to the additional orientational degree of freedom associated with the anisotropic induced magnetic moments. In Fig. 4 we provide

calculations of the distance dependence exchange energy at a fixed orientation (close to the armchair, head-to-head orientation) between AB pairs.
Additionally, to clarify these aspects, following the suggestions of the referee, we have included in the SM the schematics with the 3 possible limiting orientations, and the plot with the monotonous decay for the AB coupling at a fixed orientation (SI section 4).

[Redacted].

7.How is the consistency of the STM tip verified following hydrogen atom manipulation? The little differences in STM images between trimer and dimer configurations raise the question of tip stability. Moreover, what is the origin of the additional spectral peaks near the inelastic tunneling peaks in Fig. 4i, and should they be regarded as physically meaningful?

We verify the consistency and electronic stability of the STM tip throughout H manipulation by regular calibration measurements. Specifically, we systematically record *dI/dV* spectra on reference hydrogen configurations and confirm that these spectra remain unchanged before and after H manipulation. This procedure ensures that the electronic density of states of the tip is stable during the experiments.
While after H manipulation the topographic termination of the tip can occasionally change, resulting in slight variations in the apparent shape or contrast of hydrogen atoms in STM images, the electronic structure of the tip most often remains unaffected. Such changes may cause the tip to appear slightly broader after hydrogen removal events, but they do not influence the observed spin excitation energies. We therefore conclude that the spectral features reported here are not artifacts induced by changes in the tip apex. Throughout the experiments, we only consider tips showing the same reference spectra before and after manipulation.

Regarding the additional spectral features near the inelastic tunneling peaks in Fig. 4i, the referee is indeed correct and makes a careful observation. We have here focused our analysis on the most prominent features of the spectra. Although it is tempting to interpret the weaker nearby peaks (which are robust and not related to experimental noise) as additional spin excitations, we cannot make a clear claim, for example due to their bias asymmetry. Nevertheless, as discussed in SI Fig. 8 and 9, theoretical modeling indicates that a second spin excitation of the trimer is expected and may lie very close in energy to the primary excitation. Therefore, although we consider these additional features suggestive, we prefer not to claim them as conclusively identified spin excitations to nearly degenerate states.

Minor Suggestions:

8.Tables 1 and 2 should be organized in a systematic order (e.g., by increasing H-H distance) to improve readability.

9.All scale bars in Fig. 4 and the Supplementary Information should be visible.

We thank the reviewer for their suggestions, which we agree are necessary to improve the readability and the rigor of the paper. We have followed them and the Tables and Figure 4 are now modified accordingly.

Reviewer #2 (Remarks to the Author):

The manuscript by Vina-Bausa et al. reports on the investigation of magnetic interactions of localized spin-1/2 centers realized as chemisorbed hydrogen atoms on graphene. Scanning tunneling microscopy at low temperatures is implemented to observe the low-voltage differential conductance signatures (inelastic electron tunneling spectroscopy) giving insights into the spin states of the system. Interactions within dimers are investigated revealing pronounced exchange couplings at distances as large as 10 nm. In trimers and clusters with higher number of spins magnetic collective spin excitations are studied. Overall I find the paper interesting and appealing to the general physics community and in particular to those studying electron correlations in solids. Both experimental and theoretical data are convincing. The presentation of the manuscript is very clear and of high quality. I am fully persuaded that Nature Communications is the right journal for these findings. In particular with the current manuscript the authors establish a handy system which allows for the on-demand realization of complex spin systems. Thus, providing an important toolkit going beyond the already existing efforts. Thus I suggest publication with minor corrections.

We would like to thank the reviewer for their positive feedback.

Minor questions:

I wonder about the actual degree of control within the system. Is it possible to place the spin centers at particular graphene cites on demand or to move attached hydrogen atoms along the graphene lattice to particular positions? Or is it more a question of chance to get a desired nanostructure. In the supplementary material part the hydrogen removal process is shown, but the overall control of the actual position of the hydrogen atoms is undiscussed. Would it be possible to saturate the surface with hydrogen and to remove the hydrogen overshoot by the STM tip leaving the desired structure behind? I would happy to see the authors' ideas along these lines.

At present, H atoms are not laterally moved along the graphene lattice in a controlled manner once adsorbed. The degree of control in our system is achieved through a combination of deterministic and non-deterministic steps. Hydrogen atoms are first deposited onto a chosen graphene region with nanometer-scale spatial precision by voltage-pulse-induced deposition from the STM tip. This initial deposition step is not deterministic at the single-site level and can result in a small overshoot in the number and exact positions of adsorbed H atoms, depending on the tip apex termination. Full atomic-scale control is then obtained through selective removal of individual hydrogen atoms. By approaching the STM tip to within 1–2 Å of a chosen adsorption site, single H atoms can be reliably desorbed with essentially 100% efficiency. In this sense, the final structures are not obtained by chance but are deterministically constructed through controlled atomic removal.
In principle, the approach suggested by the referee, saturating a graphene region with hydrogen and subsequently removing hydrogen atoms to define a desired structure, is compatible with our methodology. Although in this work we have restricted our focus to studying the long-range coupling that takes place between dimers and trimers of these magnetic moments, the suggested approach would be undoubtedly useful to build larger coupled structures such as chains or rings using H atoms, or even unconventional magnetic phases as reported on *Nano Letters* ***25***, 11554-11561 (2025). We thank the reviewer for their comment and provide here an additional example (Fig. 5) where we build a 4-atom chain with a total spin S=0 (AABB configuration) starting from a region highly saturated with H.

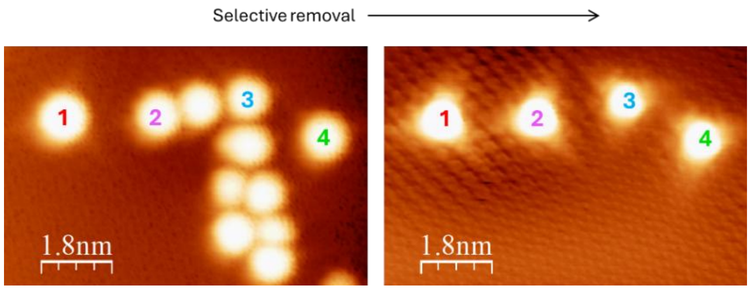

Figure 5: Assembly of a chain configuration by selective removal.

In the manuscript the authors the data on trimers (Fig. 4). I would be interested to se a more detailed discussion of the line shapes observed in the STS spectra presented in c,f,i. How the couplings are assigned departing from the experimental results?

We have modified the discussion paragraph in the section **"Spin S=1/2 trimers"** to provide a more detailed description regarding the spectral line shapes of trimers and our classification on the three different scenarios. Type III trimers correspond to configurations where the measured *dI/dV* spectra are essentially identical at all three atomic sites, indicating a regime dominated by tripartite exchange coupling. Type I trimers represent the dimerized regime, where two atoms exhibit identical spectra with spin excitations of the same energy and intensity, while the third atom does not. Finally, Type II trimers correspond to an intermediate regime, in which spin excitations are observed at all three sites but with reduced intensity on one H atom, consistent with a weakly dimerized configuration.

Additionally, we include for the reviewer here another example (Fig. 6) to illustrate how we assign pairwise coupling strengths to the spins, again based on the similarities of the *dI/dV* spectra: the presence or lack of spin excitations. In this additional example, the trimer is strongly dimerized with H1 and H2, presenting the same spin excitation features at 21 meV, while H3 is essentially decoupled, with no visible spin excitations. We assign therefore a strong antiferromagnetic coupling to the singlet 1-2, the two remaining pairwise interactions being negligible. After the atomic manipulation, we activate the coupling between the remaining AB pair. The measured spin excitation energy after the manipulation is significantly smaller (10 meV) than the one in the dimerized trimer. All this suggests that the antiferromagnetic coupling between 1 and 2 is significantly larger than the coupling corresponding to the pairs 1-3 and 2-3.

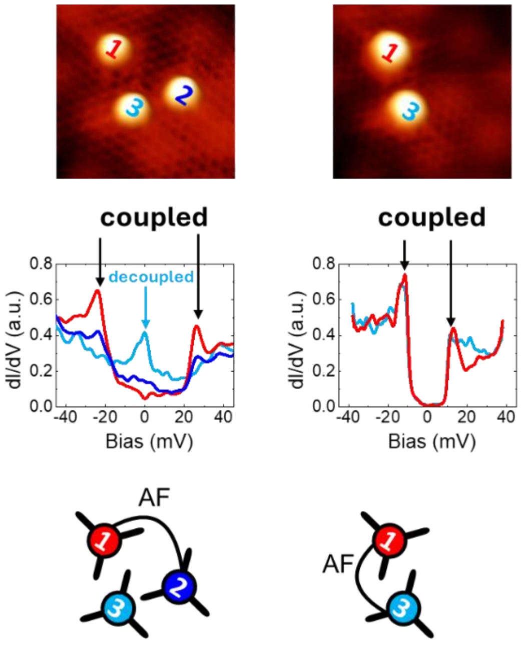

Figure 6: Additional example of a trimer configuration and its manipulation two a dimer to illustrate how the coupling strengths are assigned by comparing the *dI/dV* spectra on each of the spin sites.

Reviewer #3 (Remarks to the Author):

The authors present a detailed study of spin correlations between H atoms adsorbed on graphene. This is accomplished by means of inelastic electon tunneling spectroscopy and STS, from which the spin-triplet gap is extracted and converted into an effective exchange coupling between the spins. The control of H atoms on graphene is remarkable and the entire study is backed up by mean-field Hubbard calculations, which provides a good agreement and explanation with the results. While the results and experimental techniques are rather impressive, it is not clear to me if there are any big surprises in here - perhaps the magnitude of exchange splitting in the H-dimers, but is is not so clear what one would expect. Nevertheless, he paper is well written and comprises a nice and coherent story. It will be interesting for a broad readership and I thus recommend publication. A few comments below:

We thank the referee for their careful reading of the manuscript and for their positive appreciation of our work.

It would be useful if the results were compared with the singlet-triplet splitting of a bare H-dimer. Perhaps as a function of distance obtained by some numerical method. This would likely be a different order of magnitude but still a useful comparison.

Hydrogen pairs in our system are radically different from molecular Hydrogen (H_2). For H_2 in vacuum, the curve E(S=1)-E(S=0) vs distance is of course very different from the one obtained in our manuscript (see Eisberg, R. & Resnick, R. *Quantum Physics of Atoms, Molecules, Solids, Nuclei, and Particles*. 2nd edn (Wiley, New York, 1985)). The two main differences are range and magnitude. In the case of a bare H_2 molecule, the range of the interaction is governed by the size of the 1*s* orbital of atomic H, so that the splitting tends to zero rapidly for distances above 0.5 nm. The second difference is the energy scale, in the eV order of magnitude.

Given the very different nature of the two systems, we prefer not to include a discussion

The authors mention several exchange mechanisms at play. Direct, kinetic and RKKY. Could the authors try to quantify the contribution of these. Right now, the statement is a bit trivial because is merely states the possible mechanisms.

Quantitatively disentangling the exchange mechanisms for this system is very difficult. In the AB case we have evidence that both kinetic-like and RKKY-like contributions are present. We have performed a series of calculations for fixed distance and various strengths of the on-site Hubbard interaction (U). For U in the range 0.5t ~ 1.5t the exchange decays approximately as 1/U, which is characteristic of kinetic exchange, but it starts to increase sharply with increasing U at ~ 1.75t, which is indicative of RKKY-like contributions (see Fig. 7). From this analysis we are inclined to say that for U=t, which is the value we adopted in all the calculations shown in the manuscript, the dominant mechanism for the AB pairs is kinetic exchange, with a smaller contribution from RKKY.

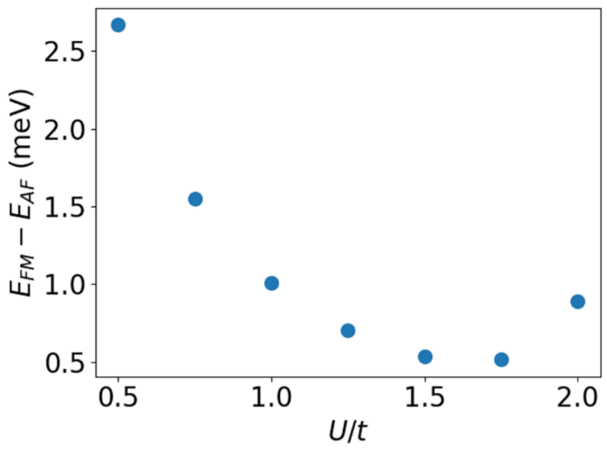

Figure 7: AB exchange as a function of Hubbard U for a distance of ~4 nm along the zigzag direction.

We also tried to disentangle the different contributions by estimating the kinetic exchange contribution using an effective Hubbard dimer model projected on the subspace of the two zero modes. We find that this contribution has the same angular dependence and very similar distance dependence as the mean-field energy differences. This confirms that kinetic exchange is the dominant mechanism for the AB pairs, but the different distance dependence indicates that the RKKY contribution is non-negligible.

For the AA dimers there is direct overlap between the wave functions of the zero modes (ZM) associated with the two H atoms. This should give a contribution to the exchange coupling that decays with distance as $1/d^2$, since the ZM's wave functions decay as 1/d. RKKY between AA pairs in graphene is expected to decay as $1/d^3$ for large distances in the case of point impurities, but even faster for magnetic moments distributed over various atomic sites. Thus, direct exchange will always dominate RKKY and it is virtually impossible to separate the two contributions. Indeed, our calculations show that the exchange between AA pairs decay as $1/d^2$ over the whole range of distances we considered, compatible with the behavior of direct exchange (see Fig. 8). Following the reviewer's comment we have added a condensed version of this discussion to the revised manuscript.

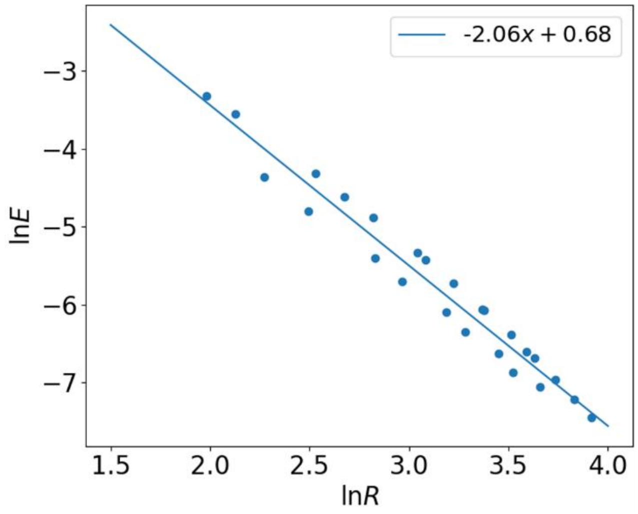

Figure 8: Exchange coupling as a function of distance for AA dimers, displaying the $1/d^2$ decay.

REVIEWERS' COMMENTS

Reviewer #1 (Remarks to the Author):

The authors have conducted a comprehensive and high-quality revision of the manuscript in response to the previous review comments, leading to a marked improvement in the manuscript quality. This work features notable innovation, systematic design and significant scientific importance, fully meeting the publication criteria of Nature Communications, and is therefore recommended for immediate acceptance.

By virtue of elaborate experimental design and in-depth theoretical analysis, the authors report the first observation of ultra-long-range spin coupling exceeding 10 nm with a coupling strength of 3 meV in graphene, breaking the spatial and energy scale limits of traditional spin systems. Combining STM atomic manipulation and IETS spectroscopic characterization, the authors realize the precise fabrication of spin dimers and trimers, achieve atomic-resolution imaging of spin excitations, and further reveal the sublattice-dependent ferromagnetic/antiferromagnetic coupling modulation as well as collective spin excitations and quantum entanglement characteristics in trimers. These findings provide a novel material platform and experimental approach for quantum magnetism and spin-based quantum computing, holding great scientific value and application potential.

The revised manuscript is excellent in supplementary data, result interpretation and figure optimization. The extensive additional experimental data and simulation results further verify the reliability of the core conclusions; the analysis of spin coupling mechanisms and spectroscopic features is more in-depth and thorough; the figures are clear and intuitive, and highly consistent with the textual discussion.

In summary, this work is characterized by novel research content, comprehensive and solid experimental data, and sufficient and persuasive argumentation. The authors have successfully completed the manuscript revision with no further revisions required. It is recommended for immediate publication in Nature Communications.

We thank the reviewer for their positive feedback and for the recommendation for publication.

Reviewer #2 (Remarks to the Author):

The manuscript by Vina-Bausa et al. reports on the investigation of magnetic interactions of localized spin-1/2 centers realized as chemisorbed hydrogen atoms on graphene. Scanning tunneling microscopy at low temperatures is implemented to observe the low-

voltage differential conductance signatures (inelastic electron tunneling spectroscopy) giving insights into the spin states of the system.

Overall I find the paper interesting and appealing to the general physics community. Both experimental and theoretical data are convincing. The authors fully addressed the questions raised by the referees and improved the manuscript accordingly. I am confident that Nature Communications is the right journal for these findings. Thus I support the acceptance of the manuscript.

We appreciate the reviewer's positive comments and their recommendation to proceed with the publication.

Reviewer #3 (Remarks to the Author):

The manuscript may be published in its current form

We thank the reviewer for their recommendation for publication.
